# Incidence and prevalence of heart failure in England: a descriptive analysis of linked primary and secondary care data – the PULSE study

**DOI:** 10.1186/s12872-023-03337-1

**Published:** 2023-07-26

**Authors:** Leana Bellanca, Stephan Linden, Ruth Farmer

**Affiliations:** 1 Boehringer Ingelheim Ltd, Bracknell, UK; 2 Boehringer Ingelheim International GmbH, Ingelheim am Rhein, Germany

**Keywords:** England, Heart Failure, Incidence, Prevalence, Real-World Evidence

## Abstract

**Background:**

Heart failure (HF) is associated with high morbidity and mortality, yet data on HF subtype (HF with reduced ejection fraction [HFrEF] and preserved ejection fraction [HFpEF]) in broad populations are lacking. Additionally, it is unknown whether current HF incidence and prevalence rates are consistent with historical data. Here, we estimate the incidence and prevalence of HF in England and describe the characteristics of patients with HF, both overall and by subtype.

**Methods:**

This was a non-interventional cohort study based on data from the UK Clinical Practice Research Datalink (CPRD), linked to Hospital Episode Statistics data and Office for National Statistics mortality data. Patients aged ≥ 18 years who were registered in the CPRD Aurum database between 1^st^ January 2015 and 31^st^ December 2019 formed the base cohort, from which patients with a recorded chronic HF diagnosis (historical or incident) from 2015–2019 contributed to the incidence and prevalence calculations.

**Results:**

The eligible denominator over the study period comprised 11,414,490 patients, from which 383,896 patients with HF were included as prevalent or incident HF cases. From 2015 to 2019, the incidence rate of newly diagnosed HF increased from 4.1/1,000 person-years to 4.9/1,000 person-years, and HF prevalence increased from 2.1% to 2.4%. Phenotype data were available for 100,224 (26.1%) patients, of which 68,780 patients had HFrEF and 31,444 had HFpEF (HFrEF/HFpEF ratio: 70.1%/29.9%). Comorbidity levels were high and broadly similar across HF subgroups.

**Conclusions:**

Primary care recording of HF subtype is suboptimal, with more than 7/10 patients with HF lacking subtype data. In patients with a recorded subtype (*n* = 100,224), a HFrEF/HFpEF ratio of 70%/30% was observed. Comorbidity levels were high regardless of subtype. Between 2015 and 2019, we observed modest but consistent increases in the incidence and prevalence of chronic HF in adults, in line with historical data.

**Supplementary Information:**

The online version contains supplementary material available at 10.1186/s12872-023-03337-1.

## Background

Heart failure (HF) is associated with reduced quality of life and high levels of morbidity and mortality [1, 2]. A previous study linking primary and secondary care data estimated the 2014 HF incidence rate in the UK to be 332 per 100,000 person-years. The absolute number of new HF cases was estimated to be 190,798, and the overall HF prevalence 920,616 cases (1.6% of the UK population) [3].

HF may present as impaired ventricular systolic or diastolic function, known as HF with reduced ejection fraction (HFrEF) and HF with preserved ejection fraction (HFpEF), respectively. Existing evidence suggests that patient demographics, causes, prognoses and responses to therapy differ by ejection fraction (EF) classification [4–7].

In the UK, most studies of HF incidence, prevalence, patient demographics, clinical outcomes and healthcare resource utilisation in broad HF populations have not investigated HF subtypes. For example, four studies investigating outcomes after an incident HF diagnosis, using data from the Clinical Practice Research Datalink (CPRD) linked to Hospital Episode Statistics (HES) data and UK Office for National Statistics (ONS) mortality data, looked at overall HF only [1, 3, 8–10].

Studies that have presented patient characteristics and outcomes by HF subtype have been conducted using small samples and single institutions that may not be representative of the entire HFrEF/HFpEF population [6, 11]. Although some studies conducted outside the UK include HF subtype data, it is not clear whether the patient demographics and their outcomes are generalisable to other countries, including England [5, 7].

In this non-interventional cohort study, we aimed to estimate current HF incidence and prevalence, and characteristics of patients with HF in England, both overall and by subtype.

## Methods

### Data sources

In September 2020, electronic health records from the CPRD Aurum database were extracted. CPRD Aurum is a longitudinal database that contains anonymised person-level primary care data on demographics, diagnoses, symptoms, prescriptions, referrals, immunisations, selected lifestyle factors, tests and results, and is representative of the English population with respect to age, gender and ethnicity [12, 13]. Data obtained from CPRD were linked to HES Admitted Patient Care data [14] and ONS mortality data [15]. Data up to March 2020 from HES and ONS were linked to 81% of the CPRD data.

### Study population

**Fig. 1:**
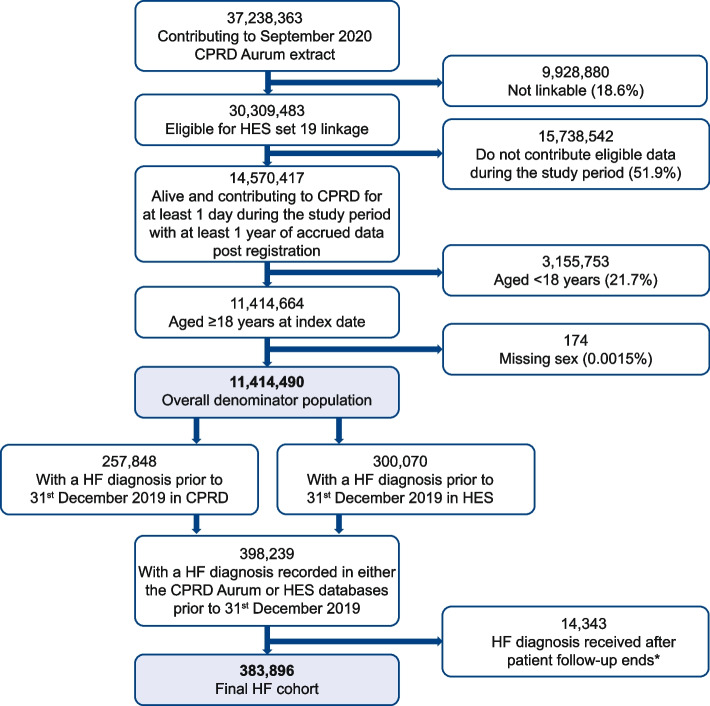
Patient flowchart for inclusion and analysis. The study period was from 1^st^ January 2015 to 31^st^ December 2019. *Patients whose HF diagnosis was recorded in the HES database only may have received their first diagnosis before 31^st^ December 2019, but after the date at which they exited follow-up from their CPRD practice (either due to de-registration or because of the date at which the practice last contributed data) and hence would not be included as patients with HF. While under follow-up, these patients are still included in the overall denominator population. *CPRD* Clinical Practice Research Datalink, *HES* Hospital Episode Statistics, *HF* heart failure

**Fig. 2:**
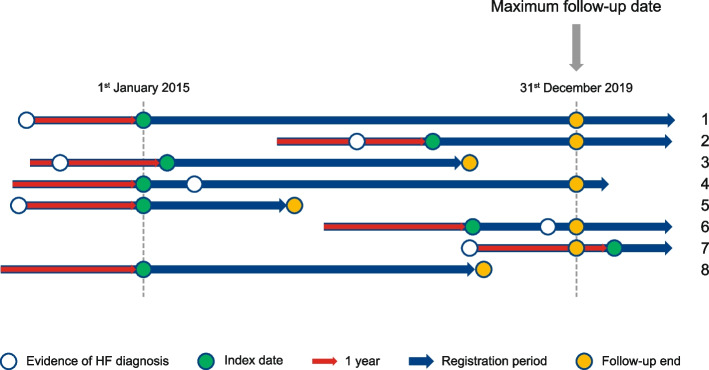
Follow-up for incidence and prevalence calculations in CPRD population. Index date = maximum (registration + 1 year, practice data quality date = 1 year, 1^st^ January 2015). Follow-up end = minimum (death, transfer out, last data collection from practice, end of study period). Patients 1, 2, 3, 5, 7: HF prevalent at index (not included in incidence calculation); Patients 4, 6, 8: no recorded HF at index (all included in incidence calculation, with patients 4 and 6 counted as having incident HF). *CPRD* Clinical Practice Research Datalink, *HF* heart failure

The source population for this study was comprised of English adults (aged ≥ 18 years). The overall denominator population consisted of all patients registered in CPRD between 1^st^ January 2015 and 31^st^ December 2019 who were aged ≥ 18 years, who had records of acceptable quality (as judged by CPRD internal quality controls), were eligible for linkage to HES and contributed at least 1 day of data during the study period (Fig. 1). As shown in Fig. 2, the index date (i.e., start of follow-up) for each patient was defined as 1 year after their registration in CPRD, no earlier than 1^st^ January 2015. The end of follow-up was defined as death, transfer out of the CPRD, last data collection from the practice, or end of study (31^st^ December 2019).

This provided a population-based cohort from which the annual prevalence of HF could be determined at five cross-sectional points (30^th^ June each calendar year), and the incidence of new HF could be determined over the duration of the study period.

### Outcomes

#### HF incidence

A HF case was considered incident if the first HF diagnosis code (see Additional file 1: Tables S1–3) for any subtype in a patient’s record fell within the study period. Data were collected for at least 12 months prior to index to ensure that incident cases identified throughout the study period were not part of an existing prevalent condition [16]. Once patients were diagnosed with HF, they no longer contributed person time to the incidence denominator (Fig. 2).

#### HF prevalence

Annual prevalence of overall HF (any HF diagnosis, see Additional file 1: Tables S1–3 for diagnostic codes) was calculated based on the number of patients in the underlying cohort with any HF diagnosis code in their record prior to the point at which prevalence was assessed.

### HF subtype categorisation

Patients with HF were categorised as having reduced or preserved EF based on whether they had the following at their index date: diagnostic code indicating an EF ≤ 40 (reduced EF) or > 40 (preserved EF) (see Additional file 1: Tables S1–3); or an EF measure recorded in CPRD in the year prior to index (≤ 40 or > 40, if available) as long as the most recent diagnostic code prior to index did not indicate inconsistent EF (as assessed by two independent colleagues with knowledge of HF in UK primary care). As not all patients in primary care have records containing an EF measure or a HF diagnosis code that differentiates between EF subtype, patients with HF without evidence of EF classification in their record at their index date were categorised as ‘unknown.’ 

Patient characteristics were recorded on the index date for patients with prevalent HF (Fig. 2: patients 1, 2, 3, 5 and 7) and on the day of diagnosis for patients with incident HF (Fig. 2: patients 4 and 6). Patient characteristics were also recorded for all patients on 30^th^ June 2019 to allow for comparison across the 5-year study period.

### Statistical analysis

The analyses performed were predominantly descriptive in nature, with no formal statistical comparisons made to infer cause-and-effect relationships. Summary estimates are presented for each outcome. Furthermore, some basic adjustments were made to account for possible confounding by age and gender in all analyses since these factors are strongly associated with HF risk. Specifically, the incidence and prevalence calculations are age- and sex-standardised to adjust for basic changes in the population demographic over time and between subtype groups (95% confidence intervals [CIs] were calculated for all prevalence and incidence estimates). Sensitivity analyses were performed to test the robustness of the results. For instance, as the study period for characterisation of patients with HF was relatively broad (2015 to 2019), we summarised the demographic and clinical characteristics of all patients with HF included in the 2019 prevalence estimate only.

This study was approved by the Independent Scientific Advisory Committee of the UK Medicines and Healthcare products Regulatory Agency on 21^st^ September 2020 (20_000051).

## Results

### Overall denominator and overall HF population

The eligible denominator CPRD adult population consisted of 11,414,490 patients. Of these, 383,896 patients with HF were identified using data from the combined databases (Fig. 1).

At study entry, 207,935 (54%) patients had a prevalent HF diagnosis. During the study period, 175,961 (46%) received an incident HF diagnosis.

### HF subtype split

From the final HF cohort of 383,896 patients, 100,224 (26.1%) had an identifiable subtype (HFrEF 68,780 patients; HFpEF 31,444 patients). As a result, 283,672 (73.9%) patients were classified as unknown.

### HF incidence

**Fig. 3:**
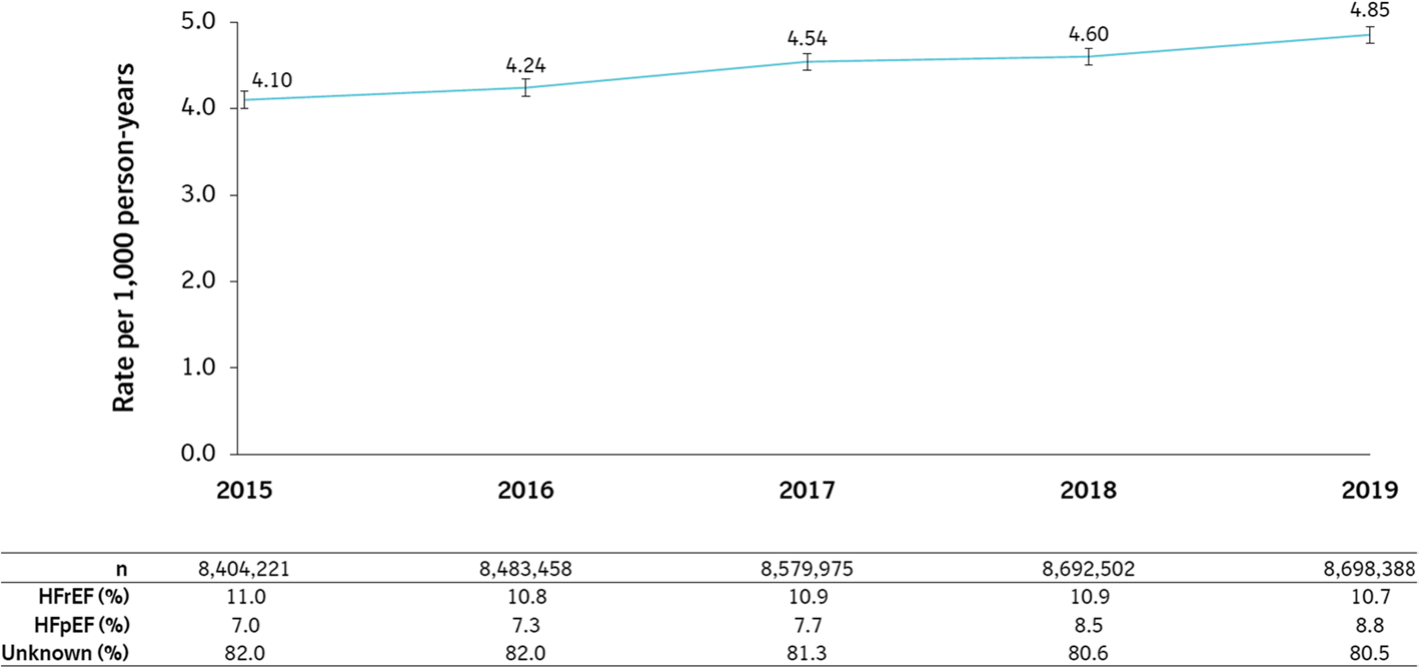
Heart failure incidence rates stratified by calendar year. Note: Calendar year was treated as time-varying via Lexis expansion for allocation of person-time to each stratum. Error bars represent 95% CI. *CI* confidence interval, *HFpEF* heart failure with preserved ejection fraction, *HFrEF* heart failure with reduced ejection fraction

**Fig. 4:**
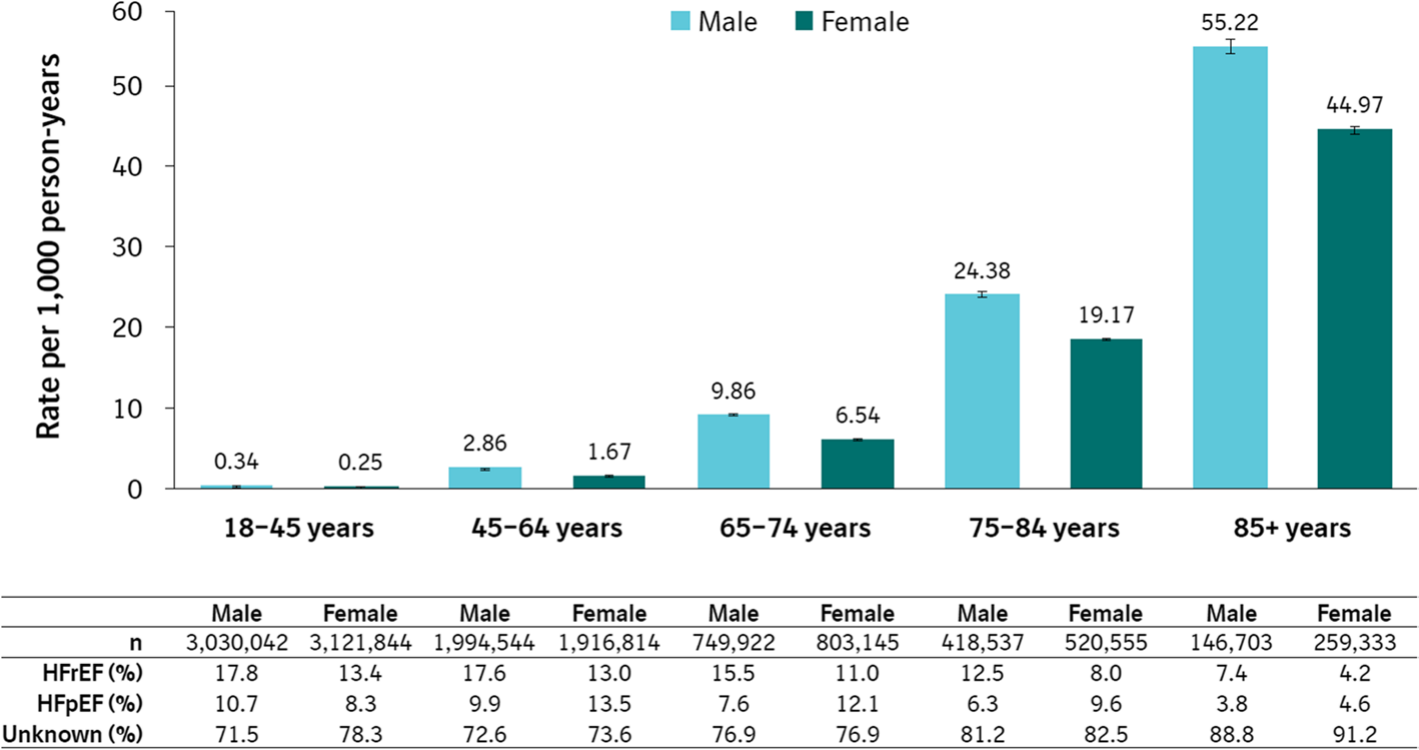
Heart failure incidence rates stratified by age and sex. Note: Age was treated as time-varying via Lexis expansion for allocation of person-time to each stratum. Error bars represent 95% CI. *CI* confidence interval, *HFpEF* heart failure with preserved ejection fraction, *HFrEF* heart failure with reduced ejection fraction

Between 2015 and 2019, excluding those with prevalent HF on their index date, data from a total of 11,210,522 patients were used to calculate HF incidence. Although 175,961 patients were identified as incident cases during follow-up, 171 patients received their first HF diagnosis on their index date and were excluded; therefore, 175,790 incident cases were observed in 39,330,587.1 person-years of follow-up (Table 1). The crude incidence rates for HF overall rose consistently, with a notable increase in HF burden from 4.10 (95% CI: 4.06–4.15) to 4.85 (95% CI: 4.80–4.90) per 1,000 patient-years between 2015 to 2019 (Fig. 3 and Table S4). Furthermore, HF incidence increased with age, peaking at 45.0 (95% CI: 44.4–45.5) and 55.2 (95% CI: 54.4–56.0) per 1,000 patient-years in female and male patients aged > 85 years, respectively (Fig. 4 and Table S4).

**Table 1:** Overall heart failure incidence rates

Overall								
	**n**	**Total PY**	**Incident HF**	**Rate per 1,000 PY**	**95% CI**	**HFrEF (%)**	**HFpEF (%)**	**% unknown**
**Total**	11,210,522	39,330,587.1	175,790	4.47	4.45–4.49	10.9	7.9	81.2

*CI* Confidence interval, *HF* Heart failure, *HFpEF* Heart failure with preserved ejection fraction, *HFrEF* Heart failure with reduced ejection fraction, *PY* Person-years

### HF prevalence

**Fig. 5:**
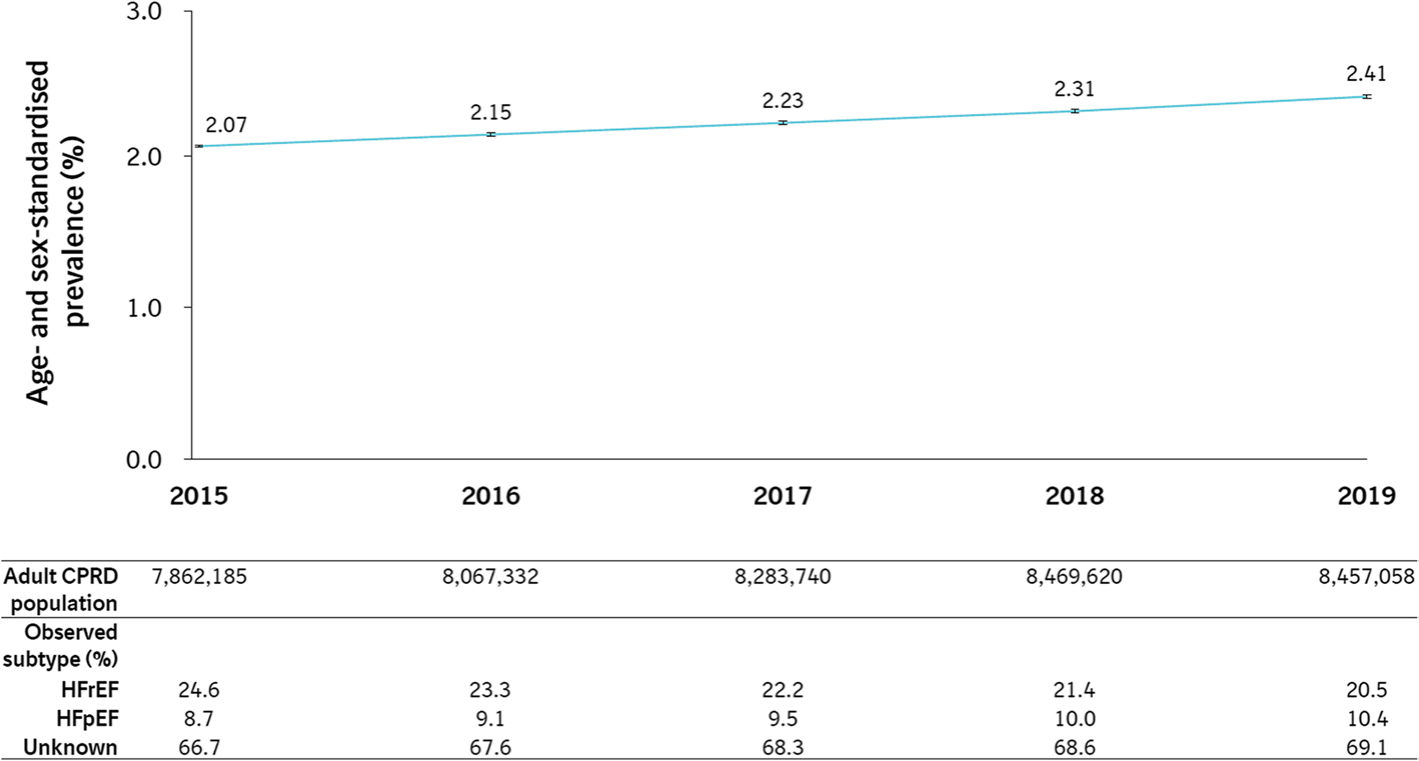
Annual prevalence of overall HF in England between 2015 and 2019 inclusive. Note: Prevalence is expressed as a percentage of the adult population (aged ≥ 18 years old). Error bars represent 95% CI. *CI* confidence interval, *CPRD* Clinical Practice Research Datalink, *HF* heart failure, *HFpEF* heart failure with preserved ejection fraction, *HFrEF* heart failure with reduced ejection fraction

Overall HF prevalence (both crude and age- and sex-adjusted) was observed to increase over time, from 2.1% in 2015 to 2.4% in 2019 (Fig. 5 and Table S5). Additionally, the proportion of HFpEF and unknown subtypes increased over time, with the proportion of HFrEF cases decreasing slightly.

### HF patient characteristics

Table 2 presents the demographic and clinical characteristics of the full HF cohort at index by subtype (*N* = 383,896). See Additional file 1: Table S6 and Table S7 for further demographic and clinical patient characteristics at index and at the 2019 prevalent cross-section.

**Table 2:** Demographic and clinical characteristics of the full HF cohort at index

	**Unknown**	**HFrEF**	**HFpEF**	**Total**
**n**	283,672	68,780	31,444	383,896
**Age at index (years)**				
Mean (SD)	76.1 (14.1)	72.2 (13.7)	71.7 (14.3)	75.0 (14.2)
**Sex**				
Female	139,125 (49.0)	24,036 (34.9)	16,287 (51.8)	179,448 (46.7)
Male	144,547 (51.0)	44,744 (65.1)	15,157 (48.2)	204,448 (53.3)
**Time since HF diagnosis (years)**				
Mean (SD)	2.3 (4.1)	3.8 (4.7)	2.2 (4.0)	2.5 (4.3)
**Number of HF hospitalisations (prior 12 months)**	30,010 (10.6)	4,048 (5.9)	811 (2.6)	34,869 (9.1)
**Ejection fraction**				
Mean (SD)	-	32.1 (8.9)	54.7 (8.3)	45.4 (14.0)
**eGFR (ml/min/1.73m**^**2**^**)**				
Mean (SD)	62.7 (21.6)	65.0 (21.5)	67.2 (20.9)	63.5 (21.6)
**eGFR < 60 ml/min/1.73cm**^**2**^	104,896 (37.0)	24,432 (35.5)	9,460 (30.1)	138,788 (36.2)
**Ever history of:**				
Hypertension	226,489 (79.8)	51,939 (75.5)	23,667 (75.3)	302,095 (78.7)
Hyperlipidemia	64,906 (22.9)	17,423 (25.3)	8,040 (25.6)	90,369 (23.5)
T2D	76,531 (27.0)	17,839 (25.9)	7,405 (23.5)	101,775 (26.5)
IHD	160,758 (56.7)	46,655 (67.8)	13,973 (44.4)	221,386 (57.7)
*MI*	69,680 (24.6)	23,273 (33.8)	4,190 (13.3)	97,143 (25.3)
*Coronary procedure*	94,732 (33.4)	33,661 (48.9)	9,317 (29.6)	137,710 (35.9)
*Other IHD*	146,871 (51.8)	41,913 (60.9)	12,437 (39.6)	201,221 (52.4)
Stroke	58,106 (20.5)	11,956 (17.4)	4,846 (15.4)	74,908 (19.5)
PAD	39,474 (13.9)	8,775 (12.8)	2,767 (8.8)	51,016 (13.3)
COPD	69,445 (24.5)	13,683 (19.9)	5,309 (16.9)	88,437 (23.0)
Atrial fibrillation	126,535 (44.6)	30,405 (44.2)	8,332 (26.5)	165,272 (43.1)
CKD	103,206 (36.4)	23,051 (33.5)	8,988 (28.6)	135,245 (35.2)
Anaemia (in the previous year)	42,165 (14.9)	5,824 (8.5)	2,783 (8.9)	50,772 (13.2)

Data are n (%) unless otherwise specified

*CKD* Chronic kidney disease, *COPD* Chronic obstructive pulmonary disease, *eGFR* Estimated glomerular filtration rate, *HF* Heart failure, *HFpEF* Heart failure with preserved ejection fraction, *HFrEF* Heart failure with reduced ejection fraction, *IHD* Ischaemic heart disease, *MI* Myocardial infarction, *PAD* Peripheral arterial disease, *SD* Standard deviation, *T2D* Type 2 diabetes

The average age of the HFrEF-coded patients at index was 72 years (73 years for prevalent, 71 years for incident), with a mean HF duration of 5.7 years in prevalent cases (median 4.5 years; interquartile range 1.9–8.4). Over half of the HFrEF-coded patients (65.1%) were males, and there was a high prevalence of hypertension (78.8% prevalent cases and 67.1% incident cases) and a history of ischaemic heart disease (IHD; 74.8% prevalent cases and 49.7% incident cases at index). Of patients with prevalent HFrEF, 27.3% had a type 2 diabetes (T2D) diagnosis at index. Drug use was broadly as anticipated, with most patients with prevalent HFrEF prescribed an angiotensin-converting enzyme inhibitor or angiotensin receptor blocker (81%) at index. Although 27% of the patients with prevalent HFrEF had been prescribed a mineralocorticoid receptor antagonist in the 6 months prior to index, the use of medications taken later in the HF treatment pathway (sacubitril/valsartan, digoxin, ivabradine and hydralazine/nitrates) was low (0.3–15.3%).

Male and female patients with HFpEF were more evenly split (48.2% male, 51.8% female) than patients with HFrEF (65.1% male, 34.9% female), although the age at index was similar for patients with HFrEF for both prevalent and incident cases. Patients with HFpEF had shorter HF duration at index (by approximately 1 year on average) and had a lower history of IHD at index compared with patients with HFrEF, but T2D prevalence was similar. Patients with prevalent HFpEF were also less likely to be on an angiotensin-converting enzyme inhibitor/angiotensin receptor blocker at index versus patients with prevalent HFrEF and to have a history of cardiac device therapy (57.7% vs 81% and 14.7% vs 24.5% respectively). Patients with HFpEF were also more likely to be of Black or South Asian ethnicity than patients with HFrEF (5.3% and 6.8% respectively vs 2.5% and 3.5% respectively).

On average, patients with an unknown subtype were older than those with a diagnosed subtype; this was particularly noticeable in the incident cohort (mean age: 76 years for unknown vs 72 years for known subtype). Additionally, fewer patients with an unknown subtype were treated with standard of care than those with a known subtype. In terms of comorbidities, the latest data, taken from the 2019 prevalent cross-section, show that a marginally lower proportion of patients with unknown subtypes had a history of IHD. However, a greater proportion had a history of other comorbidities, such as stroke, chronic obstructive pulmonary disease (COPD) and anaemia in the previous year than patients with known subtypes (see Additional file 1: Table S6 and Table S7).

### Sensitivity analyses

Results from all sensitivity analyses were broadly consistent with the main analyses, except for the estimated HFrEF/HFpEF split when considering the subgroup whose HF subtype was confirmed via a valid EF measure at index only. In this sensitivity analysis, HFpEF was the more prevalent subtype (61.5% vs 38.5%).

## Discussion

This large-scale non-interventional cohort study of English primary and secondary care data indicates a HFrEF/HFpEF ratio of 70.1%/29.9% in a population of 100,224 adults with HF. However, HF subtype data were lacking for more than two-thirds of our study population, indicating that the recording of HF subtype data in primary care is suboptimal. This could be due to inadequate use of echocardiography and other diagnostic tests and/or poor official recording of measured EF in primary care. The incidence and prevalence of chronic HF in adults have seen modest but consistent increases between 2015 and 2019: the incidence rate of newly diagnosed HF increased from 4.1/1,000 person-years to 4.9/1,000 person-years, and age- and sex-stratified HF prevalence increased from 2.1% to 2.4% [17]. Furthermore, patients with HF were observed to experience high levels of comorbidities, including IHD (approximately 2 in 3 patients), atrial fibrillation (approximately 1 in 2 patients) and T2D (approximately 1 in 3 patients). Although the prevalence of comorbidities was generally similar between patients with HFrEF and those with HFpEF, patients with HFrEF were more likely to experience IHD and atrial fibrillation (74.6% vs 51.5% and 50.9% vs 34.6% respectively).

### HF subtypes

No published literature has examined the recording of HFrEF versus HFpEF in both primary and secondary care settings in the UK. In our primary analysis, although most patients with HF had no identifiable subtype, for those who did have a subtype recorded, the estimated HFrEF/HFpEF ratio was 70.1%/29.9%. This is slightly higher, but mostly consistent, with the UK National HF Audit (2020), which reports that 64% of patients hospitalised with HF have HFrEF [18].

In the sensitivity analysis for the estimated HFrEF/HFpEF split when considering the subgroup with a valid EF measure only, HFpEF was the more prevalent subtype. Given that EF data are not available for all patients, this analysis was performed on a smaller sample size. Therefore, this discrepancy may suggest that it is more common for EF measures to be entered for patients with HFpEF.

### HF incidence and prevalence

A previous CPRD-based study estimated the prevalence of overall HF at 1.6% of the total UK population in 2014 [3] and the incidence at 3.3 per 1,000 person-years. Our prevalence estimates were higher than this; however, the denominator populations in the studies differ. In this study, the denominator was the UK adult population (≥ 18 years old). ONS 2018 population estimates suggest that 78% of the total UK population is aged ≥ 18 years old [17], so if we assume zero prevalence in the 0–17-years-old age group, our prevalence estimate based on the entire UK population would be approximately 1.6% in 2015 and 1.9% in 2019. Due to the person-year denominator, it is less straightforward to adjust our incidence estimates to reflect a similar population, but the prevalence estimate for 2015 is consistent with the previously reported CPRD-based estimate from 2014 [3]. An alternative explanation is that the present study also differs from the 2014 CPRD-based study in that a broader set of codes were used to identify patients with HF, perhaps allowing for more sensitive detection of HF cases.

The increase in HF incidence and prevalence over time observed in this study most likely reflects a true increase, but it may also partially reflect improved coding practices in primary and secondary care and/or improved HF diagnosis in the past 5 years. Nonetheless, the number of patients identified within the UK health system as having HF has increased. In summary, assuming a UK adult population of 52 million [17], the 2019 prevalence estimate equates to 1.25 million adults in the UK, the majority of which are ≥ 65 years old, as indicated by the age- and sex-stratified prevalence rates.

### Patient characteristics

Two previous CPRD-based studies have considered patient characteristics at HF diagnosis and, hence, provide a relevant comparator group for the incident HF group in this study [3, 9]. All common characteristics between these studies and the present study show good concordance, including mean/median age, male/female split, body mass index, and prevalence of comorbidities such as IHD, COPD, T2D, atrial fibrillation, hypertension and hyperlipidaemia. Both the CPRD Global Initiative for Chronic Obstructive Lung Disease and CPRD Aurum databases contain data collected from general practices; however, CPRD is based on practices across the UK whereas CPRD Aurum, which was used in this study, is larger and based on English practices only [19]. This, combined with the fact that the phenotypes of patients with HF in the UK reported in this study are similar to those reported in earlier studies, suggests that our study provides a reliable and generalisable description of English patients with HF.

Of greater interest are the characteristics of the individual subtypes: HFrEF, HFpEF and unknown. There are no studies in a broad UK patient population against which to compare our results; however, we can compare our findings to those reported by studies that used small data sets from inpatient records or specialist HF clinics. Our analysis shows that patients with an identifiable subtype were slightly younger (mean age: 72 years, median age: 74 years) than those with an unknown subtype (mean age: 76 years, median age: 79 years). The Hull LifeLab, based in northeast England, reported the mean age of 202 patients with HFrEF (defined as left ventricular EF ≤ 35%) to be 73 years [11], which is similar to our estimate. However, another study of 200 inpatients with HFrEF based in the south of England reported a median age of 82 years [6], which could be explained by the fact that these are hospitalised patients only. Our subgroup analysis by age highlighted an increased HF risk in older patients. These findings may point towards HF diagnoses being recorded but less actively treated or investigated in older patients. For example, in older patients, some HF diagnoses may be recorded as a comorbidity during a non-HF hospital admission, without full investigation or agreed follow-up.

US and European studies have previously indicated that patients with HFpEF are generally older and have a higher prevalence of comorbidities, such as hypertension and atrial fibrillation, than patients with HFrEF [20–22]. Our results show a similar mean age and similar levels of these comorbidities between the subtypes, and the differences in sex distribution and comorbidities, such as prior myocardial infarction and IHD, are consistent with these studies. Most patients with an unknown subtype were older, had a more even male/female split, and had a slightly higher prevalence of hypertension, COPD and anaemia than those with a coded subtype. They were also less likely to be prescribed HF medications. We also found that the use of medications taken later in the treatment pathway was low among patients with prevalent HFrEF. This is likely due to the lack of secondary care prescription data available, since medications taken further down the HF treatment pathway are usually initiated, and sometimes prescribed, within secondary care.

Overall, given the relative similarity of our results to those reported based on smaller UK data sources, there is reason to believe that the patients identifiable as having HFrEF or HFpEF in our study are broadly representative, in terms of their clinical and demographic characteristics, of a typical patient population treated in UK clinical practice.

### Limitations

Our study is limited by the difficulties of identifying HF and its subtypes as a condition and the patient covariates used to describe the patient population. Both rely on the presence of codes, and appropriate use of codes, in the relevant databases. The risk of classification bias is minimised by linking two databases and by studying patients with chronic HF who are likely to be monitored more frequently, and in more detail, than individuals with less severe disease. All the information on HF subtype was obtained from the primary care record, relying on a combination of recorded EF measures and diagnostic codes that distinguish between subtype. For the latter, we were unable to formally validate the codes for HFrEF and HFpEF, but they were reviewed by two independent colleagues with knowledge of HF management in UK primary care to reduce the risk of inappropriate classification. Additionally, there is a risk that patients with missing subtype information may differ to those with subtype information in terms of their disease severity and clinical characteristics, which could induce selection bias. By presenting results for individuals with unknown subtype, we were able to provide insight into this potential bias.

The results of our study suggest that 70% of patients in England with HF have HFrEF, which is slightly higher, but broadly consistent, with a 2020 UK National HF Audit (64% prevalence) [18] and a 2021 pooled analysis of > 3,500 patients from four European HF cohorts (66% prevalence) [23]. Since the coding of left ventricular systolic dysfunction is incentivised by the UK Quality and Outcomes Framework, the slightly higher prevalence of HFrEF in the present study may reflect improved implementation of this incentivisation. Data on the split between ischaemic and non-ischaemic cardiomyopathy could have strengthened the validity of our HFrEF/HFpEF estimates; however, due to concerns over the accuracy of cardiomyopathy coding in primary care, no cardiomyopathy estimates were made.

Furthermore, the CPRD database was our only source of prescription data and contains primary care data only, and therefore we have no information on prescriptions from secondary or private care. Consequently, the baseline use and ongoing rates of use of some HF medications were likely underestimated. Specific examples include sacubitril/valsartan and mineralocorticoid receptor antagonists. The former, in particular, is never initiated and infrequently prescribed in primary care, so these prescription rates should be interpreted very cautiously and lack external validity.

A further limitation of our study is the assumption that the HFrEF/HFpEF ratio of 70.1%/29.9% observed in patients with known subtypes is generalisable to patients with unknown subtypes, which may not be the case. Nonetheless, this would not affect the primary aims of this study, which were to assess the availability of HF subtype data and describe the characteristics of patients with HFrEF or HFpEF and an unknown HF subtype.

## Conclusion

Our results indicate that the recording of HF subtype is suboptimal in primary care, with most patients (73.9%) lacking subtype data. In those with a recorded subtype, our findings indicate a HFrEF/HFpEF ratio of 70%/30% and high levels of comorbidities, including IHD, atrial fibrillation and T2D in patients with both known and unknown HF subtypes. Overall, the incidence and prevalence of chronic HF in adults has seen modest, but consistent, increases between 2015 and 2019, with the largest increases in incidence seen in older age groups.

## Supplementary Information

**Additional file 1:** **Table S1. **Heart failure codes: prevalent and incidentpatients.** Table S2. **Codes to identify incident heart failure cases only. **Table S3.** ICD-10 HF codes.** Table S4.** Heart failure incidence rates stratified by calendar year and age and sex. **Table S5.** Annual prevalence of overall HF in England between 2015 and 2019 (inclusive). **Table S6.** Demographic and clinical characteristics of the full HF cohort at index. **Table S7.** Demographic and clinical characteristics: 2019 prevalent cross section.

## Data Availability

All data analysed during this study are included in this published article [and its supplementary information files]. This study is based in part on data from the Clinical Practice Research Datalink obtained under licence from the UK Medicines and Healthcare products Regulatory Agency. The data is provided by patients and collected by the NHS as part of their care and support. The interpretation and conclusions contained in this study are those of the author/s alone. The data that support the findings of this study are available from CPRD, but restrictions apply to the availability of these data, which were used under license for the current study, and so are not publicly available. Requests to access CPRD data are reviewed via the CPRD Research Data Governance (RDG) Process to ensure that the proposed research is of benefit to patients and public health. More information is available on the CPRD website: https://www.cprd.com/safeguarding-patient-data. Copyright © 2022, re-used with the permission of The Health & Social Care Information Centre. All rights reserved.

## Abbreviations

CI: Confidence interval
COPD: Chronic obstructive pulmonary disease
CPRD: Clinical Practice Research Datalink
EF: Ejection fraction
HES: Hospital Episode Statistics
HF: Heart failure
HFpEF: Heart failure with preserved ejection fraction
HFrEF: Heart failure with reduced ejection fraction
IHD: Ischaemic heart disease
ONS: UK Office for National Statistics (ONS)
T2D: Type 2 diabetes
