# Supplementary material for: Incidence and prevalence of heart failure in England: a descriptive analysis of linked primary and secondary care data – the PULSE study
Source: BMC Cardiovasc Disord. 2023 Jul 26;23:374. doi: 10.1186/s12872-023-03337-1 (PMC10373419; doi:10.1186/s12872-023-03337-1)
Supplement: Supplementary file 1 — Additional file 1: Table S1. Heart failure codes: prevalent and incidentpatients. Table S2. Codes to identify incident heart failure cases only. Table S3. ICD-10 HF codes. Table S4. Heart failure incidence rates stratified by calendar year and age and sex. Table S5. Annual prevalence of overall HF in England between 2015 and 2019 (inclusive). Table S6. Demographic and clinical characteristics of the full HF cohort at index. Table S7. Demographic and clinical characteristics: 2019 prevalent cross section. [file 12872_2023_3337_MOESM1_ESM.docx]

**Supplementary information**

**Incidence and prevalence of heart failure in England: a descriptive analysis of linked primary and secondary care data – the PULSE study**

Leana Bellanca^1^*, Stephan Linden^2^, Ruth Farmer^1^

^1^Boehringer Ingelheim Ltd., Bracknell, UK; ^2^Boehringer Ingelheim International GmbH, Ingelheim am Rhein, Germany

**Table S1.** Heart failure codes: prevalent and incident patients

| **Description** | **Subtype** | **CPRD Aurum Code** | **SNOMED description ID** |
| --- | --- | --- | --- |
| Left ventricular diastolic dysfunction | HFpEF | 1489358014 | 1489358014 |
| Heart failure with normal ejection fraction | HFpEF | 1647701000000118 | 2883808011 |
| HFNEF - heart failure with normal ejection fraction | HFpEF | 1661371000000112 | 1713091000000115 |
| Echocardiogram shows left ventricular diastolic dysfunction | HFpEF | 2159198010 | 2159198010 |
| Heart failure with preserved ejection fraction | HFpEF | 2227501000000110 | 2227501000000110 |
| Hypertrophic non-obstructive cardiomyopathy | HFpEF | 300038015 | 300038015 |
| Cardiomyopathy in Friedreich's ataxia | HFpEF | 300053011 | 300053011 |
| [X]Other hypertrophic cardiomyopathy | HFpEF | 300910013 | 350415017 |
| Diastolic dysfunction | HFpEF | 6978012 | 6978012 |
| Hypertrophic obstructive cardiomyopathy | HFpEF | 75410014 | 75410014 |
| Diastolic dysfunction | HFpEF | 939571000006115 | 939571000006115 |
| Congestive cardiomyopathy | HFrEF | 1778488011 | 1778488011 |
| Severe left ventricular systolic dysfunction | HFrEF | 1991651000006115 | 1991651000006115 |
| Echocardiogram shows left ventricular systolic dysfunction | HFrEF | 2159197017 | 2159197017 |
| Left ventricular systolic dysfunction | HFrEF | 216207010 | 216207010 |
| Ischaemic cardiomyopathy | HFrEF | 299796018 | 299796018 |
| Primary dilated cardiomyopathy | HFrEF | 300040013 | 300040013 |
| Secondary dilated cardiomyopathy | HFrEF | 34556015 | 34556015 |
| Echocardiography - poor LV function | HFrEF | 635801000006110 | 635801000006110 |
| Malignant hypertensive heart AND renal disease | Unspecified | 110659019 | 110659019 |
| Chronic cor pulmonale | Unspecified | 132655012 | 132655012 |
| Heart failure | Unspecified | 139475013 | 139475013 |
| Cardiac failure | Unspecified | 139482012 | 139482012 |
| Left ventricular failure | Unspecified | 141306010 | 141306010 |
| Cardiomyopathy | Unspecified | 142397010 | 142397010 |
| Chronic congestive heart failure | Unspecified | 147247018 | 147247018 |
| Heart failure follow-up | Unspecified | 1484917012 | 1484917012 |
| Heart failure annual review | Unspecified | 1484918019 | 1484918019 |
| Heart failure confirmed | Unspecified | 1488804017 | 1488804017 |
| Constrictive cardiomyopathy | Unspecified | 150511013 | 2532896011 |
| Heart failure lifestyle plan commenced | Unspecified | 1539381000006117 | 1539381000006117 |
| Heart failure information starter pack provided | Unspecified | 1539391000006119 | 1539391000006119 |
| Heart failure monitoring - unstable symptoms | Unspecified | 1539471000006111 | 1539471000006111 |
| Heart failure monitoring - specialist clinical needs | Unspecified | 1539481000006114 | 1539481000006114 |
| Heart failure monitoring - social issues | Unspecified | 1539491000006112 | 1539491000006112 |
| Heart failure monitoring - psychological issues | Unspecified | 1539501000006116 | 1539501000006116 |
| Heart failure monitoring - multiple readmissions | Unspecified | 1539511000006118 | 1539511000006118 |
| Heart failure monitoring - co-medications | Unspecified | 1539521000006114 | 1539521000006114 |
| Heart failure monitoring - co-morbidities | Unspecified | 1539531000006112 | 1539531000006112 |
| Heart failure monitoring - palliative care | Unspecified | 1539541000006119 | 1539541000006119 |
| Excepted heart failure quality indicators: Patient unsuitabl | Unspecified | 1561941000006119 | 1570021000000117 |
| Excepted heart failure quality indicators: Informed dissent | Unspecified | 1561951000006117 | 1570031000000115 |
| Arrhythmogenic right ventricular cardiomyopathy | Unspecified | 1656521000006113 | 1656521000006113 |
| Referral to heart failure education group declined | Unspecified | 1693911000000115 | 1693911000000115 |
| Discharge from heart failure nurse service | Unspecified | 1705341000000110 | 1705341000000110 |
| Referral to heart failure exercise programme declined | Unspecified | 1734081000000112 | 3011446018 |
| Referral to heart failure exercise programme not indicated | Unspecified | 1734161000000119 | 3011612011 |
| Has heart failure management plan | Unspecified | 1746171000000119 | 1746171000000119 |
| Emergency heart failure admission since last appointment | Unspecified | 1747691000006119 | 1747691000006119 |
| Preferred place of care for next exacerbation heart failure | Unspecified | 1784061000006118 | 1765351000000114 |
| Right ventricular failure | Unspecified | 1816101000006113 | 490972013 |
| Heart failure pathway protocol not followed | Unspecified | 1822161000006116 | 1822161000006116 |
| Heart failure clinical pathway protocol followed | Unspecified | 1824091000006119 | 1824091000006119 |
| Acute congestive heart failure | Unspecified | 18472010 | 18472010 |
| Heart failure monitoring in primary care | Unspecified | 1856361000006116 | 1856361000006116 |
| Heart failure monitoring in secondary care | Unspecified | 1856371000006111 | 1856371000006111 |
| Heart failure monitoring default | Unspecified | 1856381000006114 | 1856381000006114 |
| AURAS-AF - consider the patient to have heart failure | Unspecified | 1861731000006114 | 1861731000006114 |
| Right heart failure | Unspecified | 206703015 | 206703015 |
| Heart failure self-management plan review | Unspecified | 2115781000000114 | 2115781000000114 |
| Heart failure self-management plan agreed | Unspecified | 2117931000000116 | 2117931000000116 |
| Education about deteriorating heart failure | Unspecified | 2122191000000110 | 2122191000000110 |
| Congestive heart failure monitoring | Unspecified | 216184014 | 216184014 |
| Referral to heart failure clinic | Unspecified | 216246012 | 216246012 |
| Amyloid cardiomyopathy | Unspecified | 2196311000000112 | 2196311000000112 |
| Heart failure clinical pathway | Unspecified | 2205951000000117 | 2205951000000117 |
| Cardiac failure NOS | Unspecified | 223981000000118 | 139482012 |
| Referral to rapid access heart failure clinic | Unspecified | 2256811000000114 | 2256811000000114 |
| Left ventricular dysfunction monitoring administration | Unspecified | 226181000000110 | 1565021000000117 |
| On optimal heart failure therapy | Unspecified | 2352391000000117 | 2352391000000117 |
| Heart failure rehabilitation programme not available | Unspecified | 2405871000000117 | 2405871000000117 |
| H/O: heart failure | Unspecified | 251680018 | 251680018 |
| Left ventricular dysfunction monitoring first letter | Unspecified | 2533628012 | 2533628012 |
| Left ventricular dysfunction monitoring second letter | Unspecified | 2533629016 | 2533629016 |
| Left ventricular dysfunction monitoring third letter | Unspecified | 2533630014 | 2533630014 |
| Seen by community heart failure nurse | Unspecified | 2548316014 | 2548316014 |
| Right ventricular systolic dysfunction | Unspecified | 2548656015 | 2548656015 |
| Left ventricular dysfunction monitoring verbal invite | Unspecified | 2549089012 | 2549089012 |
| Right ventricular diastolic dysfunction | Unspecified | 2549128016 | 2549128016 |
| Admit heart failure emergency | Unspecified | 2549208013 | 3082850014 |
| Seen in heart failure clinic | Unspecified | 2549243014 | 2549243014 |
| Referral to heart failure nurse | Unspecified | 2549697018 | 2549697018 |
| New York Heart Association Classification - Class I | Unspecified | 2616470012 | 2616470012 |
| New York Heart Association Classification - Class II | Unspecified | 2616471011 | 2616471011 |
| New York Heart Association Classification - Class III | Unspecified | 2616472016 | 2616472016 |
| New York Heart Association Classification - Class IV | Unspecified | 2616473014 | 2616473014 |
| Congestive heart failure due to valvular disease | Unspecified | 2675255018 | 2675255018 |
| Left ventricular cardiac dysfunction | Unspecified | 2694523019 | 2694523019 |
| Stress cardiomyopathy | Unspecified | 2816931010 | 2816931010 |
| Takotsubo cardiomyopathy | Unspecified | 2816932015 | 2816932015 |
| Malignant hypertensive heart disease NOS | Unspecified | 299650019 | 90135019 |
| Hypertensive heart and renal disease with renal failure | Unspecified | 299673010 | 299673010 |
| Other primary cardiomyopathies | Unspecified | 300032019 | 142397010 |
| Other primary cardiomyopathy NOS | Unspecified | 300041012 | 142397010 |
| Nutritional and metabolic cardiomyopathies | Unspecified | 300042017 | 300042017 |
| Cardiomyopathy in disease EC | Unspecified | 300052018 | 300052018 |
| Cardiomyopathy in myotonic dystrophy | Unspecified | 300054017 | 300054017 |
| Dystrophic cardiomyopathy | Unspecified | 300055016 | 300055016 |
| Cardiomyopathy in diseases EC, NOS | Unspecified | 300057012 | 2575702014 |
| Cardiomyopathy due to drugs and other external agents | Unspecified | 300061018 | 350426011 |
| Cardiomyopathy NOS | Unspecified | 300062013 | 142397010 |
| Decompensated cardiac failure | Unspecified | 300179017 | 300179017 |
| Compensated cardiac failure | Unspecified | 300180019 | 300180019 |
| Acute left ventricular failure | Unspecified | 300190010 | 300190010 |
| Post cardiac operation heart failure NOS | Unspecified | 300217019 | 300214014 |
| [X]Other restrictive cardiomyopathy | Unspecified | 300911012 | 150509016 |
| [X]Other cardiomyopathies | Unspecified | 300912017 | 142397010 |
| Referred by heart failure nurse specialist | Unspecified | 303361000000111 | 303361000000111 |
| Discharge from practice nurse heart failure clinic | Unspecified | 303441000000116 | 303441000000116 |
| Did not attend practice nurse heart failure clinic | Unspecified | 303861000000118 | 303861000000118 |
| Heart failure information given to patient | Unspecified | 308011000000119 | 2451451000000116 |
| Referral to heart failure exercise programme | Unspecified | 308041000000118 | 3011242019 |
| Heart failure monitoring administration | Unspecified | 308231000000118 | 1565031000000115 |
| Heart failure review completed | Unspecified | 308261000000111 | 308261000000111 |
| Heart failure care plan discussed with patient | Unspecified | 308301000000118 | 2253411000000117 |
| Referred to heart failure education group | Unspecified | 311561000000117 | 311561000000117 |
| Congenital cardiac failure | Unspecified | 316833010 | 316833010 |
| [D]Cardiorespiratory failure | Unspecified | 317955011 | 2472092014 |
| Congestive obstructive cardiomyopathy | Unspecified | 350413012 | 350413012 |
| Heart failure as a complication of care | Unspecified | 350484012 | 350484012 |
| [X]Cardiomyopathy in metabolic diseases CE | Unspecified | 370271000006116 | 300042017 |
| Obscure African cardiomyopathy | Unspecified | 395766015 | 3448072015 |
| Nutritional and metabolic cardiomyopathy NOS | Unspecified | 395767012 | 300042017 |
| Secondary cardiomyopathy NOS | Unspecified | 395768019 | 300052018 |
| Heart failure NOS | Unspecified | 395772015 | 139475013 |
| Heart failure 6 month review | Unspecified | 404741000000119 | 404741000000119 |
| Heart failure monitoring telephone invite | Unspecified | 406801000000118 | 1570071000000118 |
| Left ventricular dysfunction monitoring telephone invite | Unspecified | 407001000000113 | 1568961000000117 |
| Heart failure monitoring verbal invite | Unspecified | 407041000000111 | 1570041000000112 |
| Heart failure monitoring first letter | Unspecified | 407061000000112 | 1569521000000114 |
| Heart failure monitoring second letter | Unspecified | 407081000000115 | 1569731000000119 |
| Heart failure monitoring third letter | Unspecified | 407101000000114 | 1569171000000116 |
| Did not attend heart failure clinic | Unspecified | 407181000000116 | 407181000000116 |
| Exception reporting: heart failure quality indicators | Unspecified | 407441000000115 | 1568511000000111 |
| Impaired left ventricular function | Unspecified | 411506018 | 411506018 |
| Arrhythmogenic right ventricular cardiomyopathy | Unspecified | 419130013 | 419130013 |
| Cardiac failure therapy | Unspecified | 451426015 | 451426015 |
| H/O: Heart failure in last year | Unspecified | 453099015 | 453099015 |
| Cardiomyopathy in Duchenne muscular dystrophy | Unspecified | 460126017 | 460126017 |
| Right ventricular failure | Unspecified | 490972013 | 490972013 |
| Congestive cardiac failure | Unspecified | 493287011 | 70653017 |
| Benign hypertensive heart disease with CCF | Unspecified | 504901000006118 | 299653017 |
| Biventricular failure | Unspecified | 510016018 | 153058012 |
| Cardiomyopathy in the puerperium | Unspecified | 537991000006111 | 103681019 |
| Congestive heart failure | Unspecified | 70653017 | 70653017 |
| Malignant hypertensive heart disease with CCF | Unspecified | 728671000006119 | 1236017010 |
| Malignant hypertensive heart disease without CCF | Unspecified | 728681000006116 | 60617018 |
| Rheumatic left ventricular failure | Unspecified | 72934016 | 72934016 |
| Hypertensive heart disease NOS with CCF | Unspecified | 741681000006111 | 107545013 |
| Hypertensive heart&renal dis wth (congestive) heart failure | Unspecified | 741701000006114 | 299672017 |
| Impaired left ventricular function | Unspecified | 784191000006110 | 411506018 |
| Hyperten heart&renal dis+both(congestv)heart and renal fail | Unspecified | 789941000006117 | 299674016 |
| Acute cor pulmonale | Unspecified | 82584011 | 82584011 |
| New York Heart Assoc classification heart failure symptoms | Unspecified | 833381000006119 | 3637504017 |
| Malignant hypertensive heart disease | Unspecified | 90135019 | 90135019 |

*CPRD* Clinical Practice Research Datalink; *HFpEF* heart failure with preserved ejection fraction; *HFrEF* heart failure with reduced ejection fraction; *LV* left ventricular.

**Table S2.** Codes to identify incident heart failure cases only

| **Description** | **Subtype** | **CPRD Aurum Code** | **SNOMED description ID** |
| --- | --- | --- | --- |
| Left ventricular diastolic dysfunction | HFpEF | 1489358014 | 1489358014 |
| Heart failure with normal ejection fraction | HFpEF | 1647701000000118 | 2883808011 |
| HFNEF - heart failure with normal ejection fraction | HFpEF | 1661371000000112 | 1713091000000115 |
| Echocardiogram shows left ventricular diastolic dysfunction | HFpEF | 2159198010 | 2159198010 |
| Heart failure with preserved ejection fraction | HFpEF | 2227501000000110 | 2227501000000110 |
| Hypertrophic non-obstructive cardiomyopathy | HFpEF | 300038015 | 300038015 |
| Cardiomyopathy in Friedreich's ataxia | HFpEF | 300053011 | 300053011 |
| [X]Other hypertrophic cardiomyopathy | HFpEF | 300910013 | 350415017 |
| Diastolic dysfunction | HFpEF | 6978012 | 6978012 |
| Hypertrophic obstructive cardiomyopathy | HFpEF | 75410014 | 75410014 |
| Diastolic dysfunction | HFpEF | 939571000006115 | 939571000006115 |
| Congestive cardiomyopathy | HFrEF | 1778488011 | 1778488011 |
| Severe left ventricular systolic dysfunction | HFrEF | 1991651000006115 | 1991651000006115 |
| Echocardiogram shows left ventricular systolic dysfunction | HFrEF | 2159197017 | 2159197017 |
| Left ventricular systolic dysfunction | HFrEF | 216207010 | 216207010 |
| Ischaemic cardiomyopathy | HFrEF | 299796018 | 299796018 |
| Primary dilated cardiomyopathy | HFrEF | 300040013 | 300040013 |
| Secondary dilated cardiomyopathy | HFrEF | 34556015 | 34556015 |
| Echocardiography - poor LV function | HFrEF | 635801000006110 | 635801000006110 |
| Malignant hypertensive heart AND renal disease | Unspecified | 110659019 | 110659019 |
| Chronic cor pulmonale | Unspecified | 132655012 | 132655012 |
| Heart failure | Unspecified | 139475013 | 139475013 |
| Cardiac failure | Unspecified | 139482012 | 139482012 |
| Left ventricular failure | Unspecified | 141306010 | 141306010 |
| Cardiomyopathy | Unspecified | 142397010 | 142397010 |
| Chronic congestive heart failure | Unspecified | 147247018 | 147247018 |
| Heart failure confirmed | Unspecified | 1488804017 | 1488804017 |
| Constrictive cardiomyopathy | Unspecified | 150511013 | 2532896011 |
| Arrhythmogenic right ventricular cardiomyopathy | Unspecified | 1656521000006113 | 1656521000006113 |
| Emergency heart failure admission since last appointment | Unspecified | 1747691000006119 | 1747691000006119 |
| Right ventricular failure | Unspecified | 1816101000006113 | 490972013 |
| Acute congestive heart failure | Unspecified | 18472010 | 18472010 |
| Right heart failure | Unspecified | 206703015 | 206703015 |
| Amyloid cardiomyopathy | Unspecified | 2196311000000112 | 2196311000000112 |
| Cardiac failure NOS | Unspecified | 223981000000118 | 139482012 |
| Seen by community heart failure nurse | Unspecified | 2548316014 | 2548316014 |
| Right ventricular systolic dysfunction | Unspecified | 2548656015 | 2548656015 |
| Right ventricular diastolic dysfunction | Unspecified | 2549128016 | 2549128016 |
| Admit heart failure emergency | Unspecified | 2549208013 | 3082850014 |
| Seen in heart failure clinic | Unspecified | 2549243014 | 2549243014 |
| New York Heart Association Classification - Class I | Unspecified | 2616470012 | 2616470012 |
| New York Heart Association Classification - Class II | Unspecified | 2616471011 | 2616471011 |
| New York Heart Association Classification - Class III | Unspecified | 2616472016 | 2616472016 |
| New York Heart Association Classification - Class IV | Unspecified | 2616473014 | 2616473014 |
| Congestive heart failure due to valvular disease | Unspecified | 2675255018 | 2675255018 |
| Left ventricular cardiac dysfunction | Unspecified | 2694523019 | 2694523019 |
| Stress cardiomyopathy | Unspecified | 2816931010 | 2816931010 |
| Takotsubo cardiomyopathy | Unspecified | 2816932015 | 2816932015 |
| Malignant hypertensive heart disease NOS | Unspecified | 299650019 | 90135019 |
| Hypertensive heart and renal disease with renal failure | Unspecified | 299673010 | 299673010 |
| Other primary cardiomyopathies | Unspecified | 300032019 | 142397010 |
| Other primary cardiomyopathy NOS | Unspecified | 300041012 | 142397010 |
| Nutritional and metabolic cardiomyopathies | Unspecified | 300042017 | 300042017 |
| Cardiomyopathy in disease EC | Unspecified | 300052018 | 300052018 |
| Cardiomyopathy in myotonic dystrophy | Unspecified | 300054017 | 300054017 |
| Dystrophic cardiomyopathy | Unspecified | 300055016 | 300055016 |
| Cardiomyopathy in diseases EC, NOS | Unspecified | 300057012 | 2575702014 |
| Cardiomyopathy due to drugs and other external agents | Unspecified | 300061018 | 350426011 |
| Cardiomyopathy NOS | Unspecified | 300062013 | 142397010 |
| Decompensated cardiac failure | Unspecified | 300179017 | 300179017 |
| Compensated cardiac failure | Unspecified | 300180019 | 300180019 |
| Acute left ventricular failure | Unspecified | 300190010 | 300190010 |
| Post cardiac operation heart failure NOS | Unspecified | 300217019 | 300214014 |
| [X]Other restrictive cardiomyopathy | Unspecified | 300911012 | 150509016 |
| [X]Other cardiomyopathies | Unspecified | 300912017 | 142397010 |
| Referred by heart failure nurse specialist | Unspecified | 303361000000111 | 303361000000111 |
| Congenital cardiac failure | Unspecified | 316833010 | 316833010 |
| [D]Cardiorespiratory failure | Unspecified | 317955011 | 2472092014 |
| Congestive obstructive cardiomyopathy | Unspecified | 350413012 | 350413012 |
| Heart failure as a complication of care | Unspecified | 350484012 | 350484012 |
| [X]Cardiomyopathy in metabolic diseases CE | Unspecified | 370271000006116 | 300042017 |
| Obscure African cardiomyopathy | Unspecified | 395766015 | 3448072015 |
| Nutritional and metabolic cardiomyopathy NOS | Unspecified | 395767012 | 300042017 |
| Secondary cardiomyopathy NOS | Unspecified | 395768019 | 300052018 |
| Heart failure NOS | Unspecified | 395772015 | 139475013 |
| Impaired left ventricular function | Unspecified | 411506018 | 411506018 |
| Arrhythmogenic right ventricular cardiomyopathy | Unspecified | 419130013 | 419130013 |
| Cardiac failure therapy | Unspecified | 451426015 | 451426015 |
| Cardiomyopathy in Duchenne muscular dystrophy | Unspecified | 460126017 | 460126017 |
| Right ventricular failure | Unspecified | 490972013 | 490972013 |
| Congestive cardiac failure | Unspecified | 493287011 | 70653017 |
| Benign hypertensive heart disease with CCF | Unspecified | 504901000006118 | 299653017 |
| Biventricular failure | Unspecified | 510016018 | 153058012 |
| Cardiomyopathy in the puerperium | Unspecified | 537991000006111 | 103681019 |
| Congestive heart failure | Unspecified | 70653017 | 70653017 |
| Malignant hypertensive heart disease with CCF | Unspecified | 728671000006119 | 1236017010 |
| Malignant hypertensive heart disease without CCF | Unspecified | 728681000006116 | 60617018 |
| Rheumatic left ventricular failure | Unspecified | 72934016 | 72934016 |
| Hypertensive heart disease NOS with CCF | Unspecified | 741681000006111 | 107545013 |
| Hypertensive heart&renal dis wth (congestive) heart failure | Unspecified | 741701000006114 | 299672017 |
| Impaired left ventricular function | Unspecified | 784191000006110 | 411506018 |
| Hyperten heart&renal dis+both(congestv)heart and renal fail | Unspecified | 789941000006117 | 299674016 |
| Acute cor pulmonale | Unspecified | 82584011 | 82584011 |
| Malignant hypertensive heart disease | Unspecified | 90135019 | 90135019 |
| Acute heart failure | Unspecified | 94251011 | 94251011 |

*CPRD* Clinical Practice Research Datalink; *HFpEF* heart failure with preserved ejection fraction; *HFrEF* heart failure with reduced ejection fraction; *LV* left ventricular.

**Table S3.** ICD-10 HF codes

| **ICD10 Code** | **Term** |
| --- | --- |
| I50.0 | Congestive heart failure |
| I50.1 | Left ventricular failure |
| I50.9 | Heart failure, unspecified |
| I42.0 | Dilated cardiomyopathy |
| I42.9 | Cardiomyopathy, unspecified |
| I11.0 | Hypertensive heart disease with (congestive) heart failure |
| I25.5 | Ischaemic Cardiomyopathy |
| I13.0 | Hypertensive heart and chronic kidney disease with heart failure and stage 1 through stage 4 chronic kidney disease, or unspecified chronic kidney disease |
| I13.2 | Hypertensive heart and chronic kidney disease with heart failure and with stage 5 chronic kidney disease, or end stage renal disease |

*HF* heart failure; *ICD-10*, International Classification of Diseases 10th Revision.

**Table S4**. Heart failure incidence rates stratified by calendar year and age and sex

| **By calendar year** | | | | | | | | |
| --- | --- | --- | --- | --- | --- | --- | --- | --- |
|  | **n** | **Total PY** | **Incident HF** | **Rate per 1,000 PY** | **95% CI** | **HFrEF (%)** | **HFpEF (%)** | **Unknown (%)** |
| **2015** | 8,404,221 | 7,708,288.8 | 31,623 | 4.10 | 4.06–4.15 | 11.0 | 7.0 | 82.0 |
| **2016** | 8,483,458 | 7,831,052.3 | 33,193 | 4.24 | 4.19–4.28 | 10.8 | 7.3 | 82.0 |
| **2017** | 8,579,975 | 7,915,342.6 | 35,948 | 4.54 | 4.49–4.59 | 10.9 | 7.7 | 81.3 |
| **2018** | 8,692,502 | 7,997,720.5 | 36,807 | 4.60 | 4.56–4.65 | 10.9 | 8.5 | 80.6 |
| **2019** | 8,698,388 | 7,878,182.9 | 38,219 | 4.85 | 4.80–4.90 | 10.7 | 8.8 | 80.5 |
| **Age- and sex-stratified** | | | | | | | | |
|  | **n** | **Total PY** | **Incident HF** | **Rate per 1,000 PY** | **95% CI** | **HFrEF (%)** | **HFpEF (%)** | **Unknown (%)** |
| **18–45 yrs, male** | 3,030,042 | 9,083,853.9 | 3,118 | 0.34 | 0.33–0.36 | 17.8 | 10.7 | 71.5 |
| **18–45 yrs, female** | 3,121,844 | 8,666,833.9 | 2,164 | 0.25 | 0.24–0.26 | 13.4 | 8.3 | 78.3 |
| **45–64 yrs, male** | 1,994,544 | 6,778,722.1 | 19,405 | 2.86 | 2.82–2.90 | 17.6 | 9.9 | 72.6 |
| **45–64 yrs, female** | 1,916,814 | 6,592,247.7 | 11,021 | 1.67 | 1.64–1.70 | 13.0 | 13.5 | 73.6 |
| **65–74 yrs, male** | 749,922 | 2,233,188.5 | 22,029 | 9.86 | 9.74–10.00 | 15.5 | 7.6 | 76.9 |
| **65–74 yrs, female** | 803,145 | 2,429,971.2 | 15,884 | 6.54 | 6.44–6.64 | 11.0 | 12.1 | 76.9 |
| **75–84 yrs, male** | 418,537 | 1,142,713.4 | 27,861 | 24.38 | 24.10–24.67 | 12.5 | 6.3 | 81.2 |
| **75–84 yrs, female** | 520,555 | 1,443,004.6 | 27,663 | 19.17 | 18.95–19.40 | 8.0 | 9.6 | 82.5 |
| **85+ yrs, male** | 146,703 | 339,066.6 | 18,722 | 55.22 | 54.43–56.01 | 7.4 | 3.8 | 88.8 |
| **85+ yrs, female** | 259,333 | 620,985.1 | 27,923 | 44.97 | 44.44–45.50 | 4.2 | 4.6 | 91.2 |

**Note:** Calendar year and age were treated as time varying via lexis expansion for allocation of person time to each stratum.

*CI* confidence interval, *HF* heart failure, *HFpEF* heart failure with preserved ejection fraction, *HFrEF* heart failure with reduced ejection fraction, *PY* person-years, *yrs* years.

**Table S5.** Annual prevalence of overall HF in England between 2015 and 2019 inclusive

| **Year** | **Adult CPRD population** | **Prevalent HF cases** | **Crude prevalence (%)** | **Age- and sex-standardised prevalence  (95% CI)** | **Observed subtype (%)** | | |
| --- | --- | --- | --- | --- | --- | --- | --- |
|  |  |  |  |  | **HFrEF** | **HFpEF** | **Unknown** |
| **2015** | 7,862,185 | 163,100 | 2.07 | 2.07%  (2.07–2.08) | 24.6 | 8.7 | 66.7 |
| **2016** | 8,067,332 | 173,279 | 2.15 | 2.15%  (2.14–2.16) | 23.3 | 9.1 | 67.6 |
| **2017** | 8,283,740 | 183,968 | 2.22 | 2.23%  (2.22–2.24) | 22.2 | 9.5 | 68.3 |
| **2018** | 8,469,620 | 194,821 | 2.30 | 2.31%  (2.3–2.32) | 21.4 | 10.0 | 68.6 |
| **2019** | 8,457,058 | 203,958 | 2.41 | 2.41%  (2.4–2.42) | 20.5 | 10.4 | 69.1 |

**Note:** Prevalence is expressed as a percentage of the adult population (aged ≥ 18 years old).

*CI* confidence interval, *CPRD* Clinical Practice Research Datalink, *HF* heart failure, *HFpEF* heart failure with preserved ejection fraction, *HFrEF* heart failure with reduced ejection fraction.

**Table S6.** Demographic and clinical characteristics of the full HF cohort at index

|  | **Unknown** | **HFrEF** | **HFpEF** | **Total** |
| --- | --- | --- | --- | --- |
| **N** | 283,672 | 68,780 | 31,444 | 383,896 |
| *Age at index (years)* | | | | |
| Mean (SD) | 76.1 (14.1) | 72.2 (13.7) | 71.7 (14.3) | 75.0 (14.2) |
| Median (IQR) | 79.0  (69.0, 86.0) | 74.0  (64.0, 82.0) | 74.0  (64.0, 82.0) | 78.0  (67.0, 85.0) |
| Min–max | 18.0–115.0 | 18.0–109.0 | 18.0–106.0 | 18.0–115.0 |
| 5th–95th percentile | 49.0–94.0 | 47.0–91.0 | 45.0–91.0 | 48.0–93.0 |
| *Sex* | | | | |
| Female | 139,125  (49.0) | 24,036  (34.9) | 16,287  (51.8) | 179,448  (46.7) |
| Male | 144,547  (51.0) | 44,744  (65.1) | 15,157  (48.2) | 204,448  (53.3) |
| *Ethnicity* | | | | |
| White | 258,645  (91.2) | 63,158  (91.8) | 26,426  (84.0) | 348,229  (90.7) |
| Black | 7,043 (2.5) | 1,705 (2.5) | 1,672 (5.3) | 10,420 (2.7) |
| South Asian | 11,740 (4.1) | 2,407 (3.5) | 2,149 (6.8) | 16,296 (4.2) |
| Mixed | 1,090 (0.4) | 265 (0.4) | 222 (0.7) | 1,577 (0.4) |
| Other | 3,168 (1.1) | 694 (1.0) | 539 (1.7) | 4,401 (1.1) |
| Unknown | 1,986 (0.7) | 551 (0.8) | 436 (1.4) | 2,973 (0.8) |
| *Geographical region* | | | | |
| East Midlands | 6,920 (2.4) | 1,864 (2.7) | 704 (2.2) | 9,488 (2.5) |
| East of England | 13,198 (4.7) | 2,714 (3.9) | 1,169 (3.7) | 17,081 (4.4) |
| London | 38,578 (13.6) | 7,967 (11.6) | 6,119 (19.5) | 52,664 (13.7) |
| North East | 12,009 (4.2) | 4,558 (6.6) | 1,166 (3.7) | 17,733 (4.6) |
| North West | 54,369 (19.2) | 14,563 (21.2) | 5,180 (16.5) | 74,112 (19.3) |
| South Central | 33,372 (11.8) | 6,872 (10.0) | 5,574 (17.7) | 45,818 (11.9) |
| South East Coast | 23,849 (8.4) | 5,348 (7.8) | 2,937 (9.3) | 32,134 (8.4) |
| South West | 41,578 (14.7) | 8,055 (11.7) | 3,379 (10.7) | 53,012 (13.8) |
| West Midlands | 48,353 (17.0) | 14,133 (20.5) | 4,639 (14.8) | 67,125 (17.5) |
| Yorkshire and the Humber | 11,419 (4.0) | 2,699 (3.9) | 576 (1.8) | 14,694 (3.8) |
| Unknown | <0.01% | <0.01% | <0.01% | <0.01% |
| *Time since HF diagnosis (years)* | | | | |
| Mean (SD) | 2.3 (4.1) | 3.8 (4.7) | 2.2 (4.0) | 2.5 (4.3) |
| Median (IQR) | 0.0 (0.0, 2.8) | 1.9 (0.0, 6.1) | 0.0 (0.0, 3.0) | 0.0 (0.0, 3.5) |
| Min–max | 0.0–55.7 | 0.0–46.0 | 0.0–56.0 | 0.0–56.0 |
| 5th–95th percentile | 0.0–12.0 | 0.0–13.6 | 0.0–10.6 | 0.0–12.2 |
| *Location of HF diagnosis** | | | | |
| Primary care |  |  |  | 144,386 (37.6) |
| Inpatient secondary care |  |  |  | 203,906 (53.1) |
| Unknown |  |  |  | 35,604 (9.3) |
| *Number of HF hospitalisations (prior 12 months)* | | | | |
|  | 30,010 (10.6) | 4,048 (5.9) | 811 (2.6) | 34,869 (9.1) |
| *Number of prior HF hospitalisations (ever)* | | | | |
| None | 232,416 (81.9) | 53,893 (78.4) | 29,351 (93.3) | 315,660 (82.2) |
| 1 | 45,122 (15.9) | 10,254 (14.9) | 1,568 (5.0) | 56,944 (14.8) |
| 2 | 4,021 (1.4) | 2,799 (4.1) | 315 (1.0) | 7,135 (1.9) |
| ≥3 | 2,113 (0.7) | 1,834 (2.7) | 210 (0.7) | 4,157 (1.1) |
| *Smoking status* | | | | |
| Never smoker | 97,245 (34.3) | 21,146 (30.7) | 12,271 (39.0) | 130,662 (34.0) |
| Ex smoker | 151,067 (53.3) | 38,615 (56.1) | 16,000 (50.9) | 205,682 (53.6) |
| Current smoker | 32,624 (11.5) | 8,737 (12.7) | 3,039 (9.7) | 44,400 (11.6) |
| Unknown | 2,736 (1.0) | 282 (0.4) | 134 (0.4) | 3,152 (0.8) |
| *BMI (kg/m^2^)* | | | | |
| Mean (SD) | 28.5 (7.0) | 28.5 (6.4) | 29.2 (6.6) | 28.6 (6.8) |
| Median (IQR) | 27.6  (23.8, 32.2) | 27.7  (24.1, 31.9) | 28.4  (24.7, 32.8) | 27.7  (24.0, 32.2) |
| Min–max | 10.0–94.7 | 10.5–75.8 | 11.0–69.9 | 10.0–94.7 |
| 5th–95th percentile | 18.8–41.2 | 19.5–40.0 | 20.0–41.2 | 19.0–41.0 |
| *BMI (kg/m^2^) WHO categories* | | | | |
| Underweight | 6,657 (2.3) | 1,443 (2.1) | 511 (1.6) | 8,611 (2.2) |
| Normal weight | 44,411 (15.7) | 13,482 (19.6) | 5,145 (16.4) | 63,038 (16.4) |
| Overweight | 49,983 (17.6) | 16,650 (24.2) | 7,201 (22.9) | 73,834 (19.2) |
| Obese | 46,822 (16.5) | 14,711 (21.4) | 7,260 (23.1) | 68,793 (17.9) |
| Very obese (40+) | 10,029 (3.5) | 2,455 (3.6) | 1,383 (4.4) | 13,867 (3.6) |
| Unknown | 125,770  (44.3) | 20,039  (29.1) | 9,944  (31.6) | 155,753  (40.6) |
| *Ejection fraction* | | | | |
| Mean (SD) | - | 32.1 (8.9) | 54.7 (8.3) | 45.4 (14.0) |
| Median (IQR) | - | 35.0  (25.0, 39.5) | 55.0  (48.0, 60.0) | 45.0  (35.0, 55.0) |
| Min–max | - | 5.0–71.0 | 33.0–86.0 | 5.0–86.0 |
| 5th–95th percentile | - | 15.0–45.0 | 44.0–70.0 | 20.0–66.0 |
| % unknown | 283,672  (100.0) | 66,600  (96.8) | 28,312  (90.0) | 378,584  (98.6) |
| *NT ProBNP* | | | | |
| Mean (SD) | 1,828.9 (2,185.8) | 1,989.3 (2,248.1) | 933.1  (1,380.4) | 1,752.3 (2,141.5) |
| Median (IQR) | 926.0  (332.0, 2,414.0) | 1,063.0  (439.0, 2,608.0) | 448.0  (159.0, 1,040.0) | 863.0  (310.0, 2,284.0) |
| Min–max | 20.0–8000.0 | 20.0–8000.0 | 20.0–8000.0 | 20.0–8000.0 |
| 5th–95th percentile | 69.0–8000.0 | 75.0–8000.0 | 41.0–3786.0 | 64.0–8000.0 |
| % unknown | 263,171  (92.8) | 65,398  (95.1) | 28,547  (90.8) | 357,116  (93.0) |
| *Systolic blood pressure* | | | | |
| Mean (SD) | 130.9 (17.9) | 127.8 (17.1) | 133.4 (17.1) | 130.5 (17.8) |
| Median (IQR) | 130.0  (120.0, 140.0) | 128.0  (117.0, 139.0) | 133.0  (122.0, 142.0) | 130.0  (120.0, 140.0) |
| Min–max | 70.0–200.0 | 70.0–200.0 | 70.0–200.0 | 70.0–200.0 |
| 5th–95th percentile | 102.0–161.0 | 100.0–157.0 | 107.0–163.0 | 102.0–160.0 |
| % unknown | 29,112 (10.3) | 5,471 (8.0) | 2,686 (8.5) | 37,269 (9.7) |
| *Dystolic blood pressure* | | | | |
| Mean (SD) | 73.5 (11.1) | 73.2 (10.8) | 74.7 (10.7) | 73.6 (11.1) |
| Median (IQR) | 73.0  (66.0, 80.0) | 72.0  (66.0, 80.0) | 75.0  (68.0, 80.0) | 73.0  (67.0, 80.0) |
| Min–max | 40.0–150.0 | 40.0–150.0 | 40.0–150.0 | 40.0–150.0 |
| 5th–95th percentile | 56.0–90.0 | 57.0–90.0 | 58.0–91.0 | 57.0–90.0 |
| % unknown | 29,980 (10.6) | 5,659 (8.2) | 2,724 (8.7) | 38,363 (10.0) |
| *Heart rate (BPM)* | | | | |
| Mean (SD) | 77.5 (16.2) | 74.6 (15.1) | 74.4 (13.8) | 76.7 (15.9) |
| Median (IQR) | 76.0  (67.0, 86.0) | 72.0  (64.0, 82.0) | 73.0  (65.0, 82.0) | 75.0  (66.0, 85.0) |
| Min–max | 30.0–160.0 | 30.0–160.0 | 30.0–160.0 | 30.0–160.0 |
| 5th–95th percentile | 56.0–106.0 | 54.0–100.0 | 55.0–98.0 | 55.0–105.0 |
| % unknown | 131,328  (46.3) | 32,360  (47.0) | 13,975  (44.4) | 177,663  (46.3) |
| *eGFR (mL/min/1.73m^2^)* | | | | |
| Mean (SD) | 62.7 (21.6) | 65.0 (21.5) | 67.2 (20.9) | 63.5 (21.6) |
| Median (IQR) | 62.8  (46.5, 79.4) | 65.2  (49.0, 81.4) | 68.3  (52.5, 83.0) | 63.8  (47.4, 80.1) |
| Min–max | 3.0–150.8 | 4.0–143.9 | 5.0–142.9 | 3.0–150.8 |
| 5th–95th percentile | 27.0–96.6 | 29.6–98.9 | 31.0–99.0 | 27.8–97.3 |
| *eGFR <60 mL/min/1.73cm^2^* | | | | |
|  | 104,896  (37.0) | 24,432  (35.5) | 9,460  (30.1) | 138,788  (36.2) |
| % unknown | 52,541 (18.5) | 9,951 (14.5) | 5,242 (16.7) | 67,734 (17.6) |
| *HF medications* | | | | |
| ACEi/ARB | 151,629 (53.5) | 50,386 (73.3) | 17,108 (54.4) | 219,123 (57.1) |
| Beta-blocker | 125,149 (44.1) | 44,888 (65.3) | 13,137 (41.8) | 183,174 (47.7) |
| Diuretic | 141,453  (49.9) | 34,021  (49.5) | 13,104  (41.7) | 188,578  (49.1) |
| MRA | 23,477 (8.3) | 14,295 (20.8) | 2,232 (7.1) | 40,004 (10.4) |
| Sacubitril/valsartan | 77 (0.0) | 174 (0.3) | <10 | 258 (0.1) |
| Ivabradine | 2,198 (0.8) | 1,602 (2.3) | 208 (0.7) | 4,008 (1.0) |
| Digoxin | 25,842 (9.1) | 8,434 (12.3) | 1,321 (4.2) | 35,597 (9.3) |
| Hydralazine/nitrate | 404 (0.1) | 298 (0.4) | 38 (0.1) | 740 (0.2) |
| At least 1 HF medication (excluding diuretics) | 200,993  (70.9) | 58,627  (85.2) | 22,067  (70.2) | 281,687  (73.4) |
| *Other CV medications* | | | | |
| Statins | 152,474  (53.8) | 42,853  (62.3) | 17,131  (54.5) | 212,458  (55.3) |
| Antiplatelet therapy | 112,969  (39.8) | 31,285  (45.5) | 11,275  (35.9) | 155,529  (40.5) |
| Oral anticoagulants | 77,246  (27.2) | 23,012  (33.5) | 6,008  (19.1) | 106,266  (27.7) |
| Calcium channel blockers | 81,386  (28.7) | 13,732  (20.0) | 10,325  (32.8) | 105,443  (27.5) |
| *Ever history of:* | | | | |
| Fitted cardiac device | 38,441 (13.6) | 14,891 (21.7) | 3,539 (11.3) | 56,871 (14.8) |
| Hypertension | 226,489  (79.8) | 51,939  (75.5) | 23,667  (75.3) | 302,095 (78.7) |
| Hyperlipidemia | 64,906 (22.9) | 17,423 (25.3) | 8,040 (25.6) | 90,369 (23.5) |
| T2D | 76,531  (27.0) | 17,839  (25.9) | 7,405  (23.5) | 101,775  (26.5) |
| T1D | 3,858 (1.4) | 826 (1.2) | 298 (0.9) | 4,982 (1.3) |
| IHD | 160,758  (56.7) | 46,655  (67.8) | 13,973  (44.4) | 221,386  (57.7) |
| *MI* | 69,680 (24.6) | 23,273 (33.8) | 4,190 (13.3) | 97,143 (25.3) |
| *Coronary procedure* | 94,732  (33.4) | 33,661  (48.9) | 9,317  (29.6) | 137,710  (35.9) |
| *Other IHD* | 146,871  (51.8) | 41,913  (60.9) | 12,437  (39.6) | 201,221  (52.4) |
| Stroke | 58,106 (20.5) | 11,956 (17.4) | 4,846 (15.4) | 74,908 (19.5) |
| PAD | 39,474 (13.9) | 8,775 (12.8) | 2,767 (8.8) | 51,016 (13.3) |
| COPD | 69,445 (24.5) | 13,683 (19.9) | 5,309 (16.9) | 88,437 (23.0) |
| Atrial fibrillation | 126,535  (44.6) | 30,405  (44.2) | 8,332  (26.5) | 165,272  (43.1) |
| CKD | 103,206  (36.4) | 23,051  (33.5) | 8,988  (28.6) | 135,245  (35.2) |
| Anaemia (in the previous year) | 42,165 (14.9) | 5,824 (8.5) | 2,783 (8.9) | 50,772 (13.2) |
| *ACEi/ARB and beta-blocker substance breakdown:* | | | | |
| ACEi | | | | |
| *Enalapril* | 4,297 (4) | 971 (2.6) | 458 (4.1) | 5,726 (3.6) |
| *Lisinopril* | 17,196  (15.9) | 4,520  (11.9) | 1,966  (17.5) | 23,682  (15.1) |
| *Perindopril* | 12,903  (12) | 4,788  (12.6) | 1,349  (12) | 19,040  (12.1) |
| *Ramipril* | 72,735  (67.4) | 27,442  (72.4) | 7,394  (65.6) | 107,571 (68.5) |
| *Other* | 716 (0.7) | 181 (0.5) | 97 (0.9) | 994 (0.6) |
| ARB | | | | |
| *Candesartan* | 17,305  (37.2) | 6,425  (47.8) | 2,361  (38) | 26,091  (39.5) |
| *losartan* | 20,509  (44.1) | 5,152  (38.3) | 2,684  (43.2) | 28,345  (42.9) |
| *Irbesartan* | 5,267 (11.3) | 1,100 (8.2) | 699 (11.3) | 7,066 (10.7) |
| *Other* | 3,389 (7.3) | 768 (5.7) | 465 (7.5) | 4,622 (7) |
| Beta blockers | | | | |
| *Bisoprolol* | 100,248  (80.1) | 38,167  (85) | 10,366  (78.9) | 148,781  (81.2) |
| *Atenolol* | 17,054  (13.6) | 2,535  (5.6) | 2,019  (15.4) | 21,608  (11.8) |
| *Other* | 7,847 (6.3) | 4,186 (9.3) | 752 (5.7) | 12,785 (7) |

Data are n (%) unless otherwise specified. *Displayed overall only because subtype information is only available from primary care sources, which will bias the result towards primary care diagnosis.  
*ACEi*, angiotensin-converting enzyme inhibitor, *ARB* angiotensin-receptor blocker, *BMI* body mass index, *BPM* beats per minute, *CKD* chronic kidney disease, *COPD* chronic obstructive pulmonary disease, *CV* cardiovascular, *eGFR* estimated glomerular filtration rate, *HF* heart failure, *HFpEF* heart failure with preserved ejection fraction, *HFrEF* heart failure with reduced ejection fraction, *IHD* ischaemic heart disease, *IQR* interquartile range, *max* maximum, *MI* myocardial infarction, *min* minimum, *MRA* mineralocorticoid receptor antagonist, *NT ProBNP*, N-terminal pro B-type natriuretic peptide, *PAD* peripheral arterial disease, *SD* standard deviation, *T1D* type 1 diabetes, *T2D* type 2 diabetes, *WHO* World Health Organization.

**Table S7.** Demographic and clinical characteristics: 2019 prevalent cross section

|  | **Unknown** | **HFrEF** | **HFpEF** | **Total** |  |
| --- | --- | --- | --- | --- | --- |
| **n** | 125,088 | 54,878 | 23,993 | 203,959 |  |
| *Age at index (years)* | | | | |  |
| Mean (SD) | 74.8 (14.2) | 72.8 (13.2) | 73.2 (13.7) | 74.1 (13.9) |  |
| Median (IQR) | 77.0  (67.0, 85.0) | 75.0  (65.0, 83.0) | 75.0  (65.0, 83.0) | 76.0  (66.0, 84.0) |  |
| Min–max | 18.0–110.0 | 19.0–106.0 | 18.0–106.0 | 18.0–110.0 |  |
| 5th–95th percentile | 48.0–93.0 | 49.0–91.0 | 48.0–91.0 | 48.0–92.0 |  |
| *Sex* | | | | |  |
| Female | 59,212 (47.3) | 18,832 (34.3) | 12,456 (51.9) | 90,500 (44.4) |  |
| Male | 65,876  (52.7) | 36,046  (65.7) | 11,537  (48.1) | 113,459 (55.6) |  |
| *Ethnicity* | | | | |  |
| White | 112,515 (89.9) | 49,975  (91.1) | 19,923  (83.0) | 182,413 (89.4) |  |
| Black | 3,418 (2.7) | 1,433 (2.6) | 1,355 (5.6) | 6,206 (3.0) |  |
| South Asian | 5,989 (4.8) | 2,117 (3.9) | 1,768 (7.4) | 9,874 (4.8) |  |
| Mixed | 589 (0.5) | 222 (0.4) | 174 (0.7) | 985 (0.5) |  |
| Other | 1,562 (1.2) | 630 (1.1) | 419 (1.7) | 2,611 (1.3) |  |
| Unknown | 1,015 (0.8) | 501 (0.9) | 354 (1.5) | 1,870 (0.9) |  |
| *Geographical region* | | | | |  |
| East Midlands | 2,719 (2.2) | 1,457 (2.7) | 477 (2.0) | 4,653 (2.3) |  |
| East of England | 5,516 (4.4) | 2,123 (3.9) | 854 (3.6) | 8,493 (4.2) |  |
| London | 17,578 (14.1) | 6,584 (12.0) | 4,938 (20.6) | 29,100 (14.3) |  |
| North East | 5,593 (4.5) | 3,346 (6.1) | 871 (3.6) | 9,810 (4.8) |  |
| North West | 24,534 (19.6) | 12,011 (21.9) | 3,936 (16.4) | 40,481 (19.8) |  |
| South Central | 14,656 (11.7) | 5,844 (10.6) | 4,431 (18.5) | 24,931 (12.2) |  |
| South East Coast | 10,165 (8.1) | 3,964 (7.2) | 2,021 (8.4) | 16,150 (7.9) |  |
| South West | 19,000 (15.2) | 6,420 (11.7) | 2,557 (10.7) | 27,977 (13.7) |  |
| West Midlands | 20,706 (16.6) | 11,040 (20.1) | 3,508 (14.6) | 35,254 (17.3) |  |
| Yorkshire and the Humber | 4,621 (3.7) | 2,089 (3.8) | 400 (1.7) | 7,110 (3.5) |  |
| *Time since HF diagnosis (years)* | | | | |  |
| Mean (SD) | 5.2 (5.1) | 6.1 (5.2) | 4.8 (4.7) | 5.4 (5.1) |  |
| Median (IQR) | 3.5 (1.4, 7.2) | 4.6 (2.1, 8.8) | 3.4 (1.4, 6.7) | 3.7 (1.5, 7.5) |  |
| Min–max | 0.0–54.5 | 0.0–48.5 | 0.0–60.5 | 0.0–60.5 |  |
| 5th–95th percentile | 0.2–16.5 | 0.4–16.6 | 0.3–14.5 | 0.3–16.3 |  |
| *Location of HF diagnosis** | | | | |  |
| Primary care |  |  |  | 90,464 (44.4) |  |
| Inpatient secondary care |  |  |  | 93,878 (46.0) |  |
| Unknown |  |  |  | 19,617 (9.6) |  |
| *Hospitalisation for HF in prior 12 months* | | | | |  |
|  | 6,369 (5.1) | 3,544 (6.5) | 825 (3.4) | 10,738 (5.3) |  |
| *Number of prior hospitalisations for HF (ever)* | | | | |  |
| None | 101,463 (81.1) | 39,160  (71.4) | 21,376  (89.1) | 161,999 (79.4) |  |
| 1 | 18,651 (14.9) | 10,341 (18.8) | 1,832 (7.6) | 30,824 (15.1) |  |
| 2 | 3,304 (2.6) | 3,224 (5.9) | 454 (1.9) | 6,982 (3.4) |  |
| ≥3 | 1,670 (1.3) | 2,153 (3.9) | 331 (1.4) | 4,154 (2.0) |  |
| *Smoking status* | | | | |  |
| Never smoker | 42,251 (33.8) | 16,804 (30.6) | 9,268 (38.6) | 68,323 (33.5) |  |
| Ex-smoker | 69,809  (55.8) | 31,825  (58.0) | 12,739  (53.1) | 114,373 (56.1) |  |
| Current smoker | 12,387 (9.9) | 6,173 (11.2) | 1,942 (8.1) | 20,502 (10.1) |  |
| Unknown | 641 (0.5) | 76 (0.1) | 44 (0.2) | 761 (0.4) |  |
| *BMI (kg/m^2^)* | | | | |  |
| Mean (SD) | 29.4 (7.0) | 29.1 (6.5) | 29.9 (6.7) | 29.3 (6.8) |  |
| Median (IQR) | 28.4  (24.6, 33.0) | 28.2  (24.7, 32.5) | 29.1  (25.2, 33.6) | 28.4  (24.7, 33.0) |  |
| Min–max | 10.4–89.6 | 11.3–116.6 | 11.0–68.5 | 10.4–116.6 |  |
| 5th–95th percentile | 19.6–42.0 | 20.0–40.8 | 20.5–42.1 | 19.8–41.7 |  |
| *BMI (kg/m^2^) WHO categories* | | | | |  |
| Underweight | 1,787 (1.4) | 659 (1.2) | 213 (0.9) | 2,659 (1.3) |  |
| Normal weight | 14,310 (11.4) | 7,176 (13.1) | 2,569 (10.7) | 24,055 (11.8) |  |
| Overweight | 18,989 (15.2) | 9,962 (18.2) | 3,869 (16.1) | 32,820 (16.1) |  |
| Obese | 19,756 (15.8) | 9,727 (17.7) | 4,436 (18.5) | 33,919 (16.6) |  |
| Very obese (40+) | 4,417 (3.5) | 1,706 (3.1) | 912 (3.8) | 7,035 (3.4) |  |
| Missing | 65,829  (52.6) | 25,648  (46.7) | 11,994  (50.0) | 103,471 (50.7) |  |
| *Ejection fraction* | | | | |  |
| Mean (SD) | - | 32.7 (8.6) | 54.2 (8.4) | 44.9 (13.6) |  |
| Median (IQR) | - | 35.0 (28.0, 39.0) | 55.0 (47.0, 60.0) | 45.0 (35.0, 55.0) |  |
| Min–max | - | 5.0–71.0 | 15.0–100.0 | 5.0–100.0 |  |
| 5th–95th percentile | - | 17.0–45.0 | 44.0–70.0 | 20.0–66.0 |  |
| % missing | 125,088 (100.0) | 53,360  (97.2) | 21,987  (91.6) | 200,435 (98.3) |  |
| *NT ProBNP* | | | | |  |
| Mean (SD) | 1431.5 (1856.9) | 1930.6 (2221.3) | 998.0 (1459.6) | 1486.9 (1921.8) |  |
| Median (IQR) | 684.5  (233.0, 1817.0) | 1045.0  (374.0, 2515.0) | 454.5  (158.0, 1137.0) | 709.0  (241.0, 1885.0) |  |
| Min–max | 20.0–8000.0 | 20.0–8000.0 | 20.0–8000.0 | 20.0–8000.0 |  |
| 5th–95th percentile | 56.0–6003.0 | 73.0–8000.0 | 46.0–4062.0 | 56.0–6460.0 |  |
| % missing | 110,106 (88.0) | 49,007 (89.3) | 20,361  (84.9) | 179,474 (88.0) |  |
| *Systolic blood pressure* | | | | |  |
| Mean (SD) | 130.1 (16.7) | 126.3 (16.5) | 132.0 (16.2) | 129.3 (16.7) |  |
| Median (IQR) | 130.0  (120.0, 140.0) | 126.0  (115.0, 137.0) | 132.0  (121.0, 140.0) | 130.0  (119.0, 140.0) |  |
| Min–max | 70.0–200.0 | 70.0–200.0 | 70.0–200.0 | 70.0–200.0 |  |
| 5th–95th percentile | 104.0–159.0 | 100.0–152.0 | 106.0–160.0 | 102.0–158.0 |  |
| % missing | 15,301 (12.2) | 5,267 (9.6) | 2,855 (11.9) | 23,423 (11.5) |  |
| *Dystolic blood pressure* | | | | |  |
| Mean (SD) | 73.3 (10.4) | 72.4 (10.3) | 74.0 (10.2) | 73.1 (10.4) |  |
| Median (IQR) | 73.0  (66.0, 80.0) | 72.0  (65.0, 80.0) | 74.0  (68.0, 80.0) | 73.0  (66.0, 80.0) |  |
| Min–Max | 40.0–149.0 | 40.0–140.0 | 40.0–141.0 | 40.0–149.0 |  |
| 5th–95th percentile | 58.0–90.0 | 57.0–89.0 | 58.0–90.0 | 57.0–90.0 |  |
| % missing | 15,183 (12.1) | 5,209 (9.5) | 2,827 (11.8) | 23,219 (11.4) |  |
| *Heart rate (BPM)* | | | | |  |
| Mean (SD) | 74.4 (13.6) | 72.4 (13.1) | 73.6 (13.1) | 73.7 (13.4) |  |
| Median (IQR) | 73.0 (65.0, 82.0) | 71.0  (63.0, 80.0) | 72.0  (64.0, 81.0) | 72.0  (64.0, 81.0) |  |
| Min–max | 30.0–160.0 | 30.0–160.0 | 30.0–160.0 | 30.0–160.0 |  |
| 5th–95th percentile | 55.0–98.0 | 54.0–95.0 | 55.0–96.0 | 55.0–97.0 |  |
| *% missing* | 42,749 (34.2) | 17,143 (31.2) | 7,842 (32.7) | 67,734 (33.2) |  |
| *eGFR (mL/min/1.73m^2^)* | | | | |  |
| Mean (SD) | 63.2 (21.4) | 63.5 (21.3) | 64.9 (21.0) | 63.5 (21.4) |  |
| Median (IQR) | 63.4  (47.3, 79.8) | 63.4  (47.5, 79.8) | 65.7  (49.5, 81.2) | 63.7  (47.6, 80.0) |  |
| Min–max | 3.0–146.4 | 4.0–201.0 | 4.0–133.7 | 3.0–201.0 |  |
| 5th–95th percentile | 28.0–96.9 | 29.0–97.5 | 29.3–96.9 | 28.4–97.1 |  |
| *eGFR <60 mL/min/1.73cm^2^* | | | | |  |
|  | 45,918  (15.05) | 21,419  (39.0) | 8,040  (33.5) | 75,377  (19.6) |  |
| % missing | 22,003 (17.6) | 6,708 (12.2) | 4,235 (17.7) | 32,946 (16.2) |  |
| *HF medications n (%)* | | | | |  |
| ACEi/ARB | 70,604 (56.4) | 41,989 (76.5) | 12,999 (54.2) | 125,592 (61.6) |  |
| Beta Blocker | 67,528 (54.0) | 42,009 (76.5) | 11,404 (47.5) | 120,941 (59.3) |  |
| Diuretic | 58,963 (47.1) | 26,816 (48.9) | 10,010 (41.7) | 95,789 (47.0) |  |
| MRA | 15,036 (12.0) | 16,285 (29.7) | 2,349 (9.8) | 33,670 (16.5) |  |
| Sacubitril/valsartan | 672 (0.5) | 1,958 (3.6) | 78 (0.3) | 2,708 (1.3) |  |
| Ivabradine | 1,484 (1.2) | 1,832 (3.3) | 186 (0.8) | 3,502 (1.7) |  |
| Digoxin | 12,004 (9.6) | 6,684 (12.2) | 1,171 (4.9) | 19,859 (9.7) |  |
| Hydralazine/nitrate | 310 (0.2) | 345 (0.6) | 64 (0.3) | 719 (0.4) |  |
| At least 1 HF medication (excl. diuretics) | 96,039 (76.8) | 50,528 (92.1) | 17,688 (73.7) | 164,255 (80.5) |  |
| *Other CV medications n (%)* | | | | |  |
| Statins | 73,087 (58.4) | 35,868 (65.4) | 13,660 (56.9) | 122,615 (60.1) |  |
| Antiplatelet therapy | 47,030 (37.6) | 22,596 (41.2) | 7,740 (32.3) | 77,366 (37.9) |  |
| Oral anticoagulants | 45,441 (36.3) | 23,548 (42.9) | 6,693 (27.9) | 75,682 (37.1) |  |
| Calcium channel blockers | 31,919 (25.5) | 9,157 (16.7) | 7,619 (31.8) | 48,695 (23.9) |  |
| *Ever history of:* | | | | |  |
| Fitted cardiac device | 21,174 (16.9) | 14,221 (25.9) | 3,241 (13.5) | 38,636 (18.9) |  |
| Hypertension | 102,569 (82.0) | 43,967 (80.1) | 19,154 (79.8) | 165,690 (81.2) |  |
| Hyperlipidemia | 31,277 (25.0) | 14,547 (26.5) | 6,643 (27.7) | 52,467 (25.7) |  |
| T2DM | 37,206 (29.7) | 16,009 (29.2) | 6,577 (27.4) | 59,792 (29.3) |  |
| T1DM | 1,869 (1.5) | 660 (1.2) | 262 (1.1) | 2,791 (1.4) |  |
| IHD | 79,654 (63.7) | 40,948 (74.6) | 12,347 (51.5) | 132,949 (65.2) |  |
| *MI* | 32,253 (25.8) | 18,975 (34.6) | 3,431 (14.3) | 54,659 (26.8) |  |
| *Coronary procedure* | 54,503 (43.6) | 31,501 (57.4) | 8,390 (35.0) | 94,394 (46.3) |  |
| *Other IHD* | 73,620 (58.9) | 36,999 (67.4) | 11,134 (46.4) | 121,753 (59.7) |  |
| Stroke | 23,486 (18.8) | 8,776 (16.0) | 3,567 (14.9) | 35,829 (17.6) |  |
| PAD | 18,590 (14.9) | 7,967 (14.5) | 2,625 (10.9) | 29,182 (14.3) |  |
| COPD | 33,327 (26.6) | 12,747 (23.2) | 5,007 (20.9) | 51,081 (25.0) |  |
| Atrial fibrillation | 59,150 (47.3) | 27,946 (50.9) | 8,312 (34.6) | 95,408 (46.8) |  |
| CKD | 37,274 (29.8) | 14,532 (26.5) | 6,214 (25.9) | 58,020 (28.4) |  |
| Anaemia (in the previous year) | 15,385 (12.3) | 5,538 (10.1) | 2,507 (10.4) | 23,430 (11.5) |  |
| *ACEi/ARB and beta-blocker substance breakdown:* | | | | |  |
| ACEi | | | | |  |
| Enalapril | 1,649 (2.8) | 470 (2) | 227 (3.2) | 2,346 (2.7) |  |
| Lisinopril | 6,615 (11.4) | 2,171 (9.4) | 1,089 (15.4) | 9,875 (11.2) |  |
| Perindopril | 5,980 (10.3) | 2,635 (11.4) | 765 (10.8) | 9,380 (10.6) |  |
| Ramipril | 43,482 (75.1) | 17,784 (76.9) | 4,976 (70.2) | 66,242 (75.2) |  |
| Other | 155 (0.3) | 54 (0.2) | 33 (0.5) | 242 (0.3) |  |
| ARB | | | | |  |
| Candesartan | 10,658 (41.6) | 4,640 (50.9) | 1,717 (39.4) | 17,015 (43.5) |  |
| Losartan | 11,499 (44.8) | 3,543 (38.9) | 1,960 (45) | 17,002 (43.5) |  |
| Irbesartan | 2,208 (8.6) | 576 (6.3) | 422 (9.7) | 3,206 (8.2) |  |
| Other | 1285 (5) | 360 (3.9) | 257 (5.9) | 1,902 (4.9) |  |
| *Beta-blockers* | | | | |  |
| Bisoprolol | 70,585  (88.3) | 27,765  (88.6) | 8,274  (85.5) | 106,624  (88.2) |  |
| Atenolol | 5,028 (6.3) | 921 (2.9) | 924 (9.6) | 6,873 (5.7) |  |
| Other | 4,327 (5.4) | 2,642 (8.4) | 475 (4.9) | 7,444 (6.2) |  |

Data are n (%) unless otherwise specified. *Displayed overall only because subtype information is only available from primary care sources, which will bias the result towards primary care diagnosis.  
*ACEi*, angiotensin-converting enzyme inhibitor, *ARB* angiotensin-receptor blocker, *BMI* body mass index, *BPM* beats per minute, *CKD* chronic kidney disease, *COPD* chronic obstructive pulmonary disease, *CV* cardiovascular, *eGFR* estimated glomerular filtration rate, *HF* heart failure, *HFpEF* heart failure with preserved ejection fraction, *HFrEF* heart failure with reduced ejection fraction, *IHD* ischaemic heart disease, *IQR* interquartile range, *max* maximum, *MI* myocardial infarction, *min* minimum, *MRA* mineralocorticoid receptor antagonist, *NT ProBNP*, N-terminal pro B-type natriuretic peptide, *PAD* peripheral arterial disease, *SD* standard deviation, *T1DM* type 1 diabetes mellitus, *T2DM* type 2 diabetes mellitus, *WHO* World Health Organization.
